# Prematurely Aged Human Microglia Exhibit Impaired Stress Response and Defective Nucleocytoplasmic Shuttling of ALS Associated FUS

**DOI:** 10.1111/acel.70232

**Published:** 2025-09-19

**Authors:** Christiane Hartmann, Christina Haß, Muriel Knobloch, Israel Barrantes, Laura Fumagalli, Jessie Premereur, Franz Markert, Maite Peters, Georgia Koromila, Alexander Hartmann, Kathrin Jäger, Jette Abel, Renzo Mancuso, Alexander Storch, Michael Walter, Georg Fuellen, Andreas Hermann

**Affiliations:** ^1^ Translational Neurodegeneration Section “Albrecht Kossel”, Department of Neurology Rostock University Medical Center Rostock Germany; ^2^ Institute for Biostatistics and Informatics in Medicine and Aging Research Rostock University Medical Center Rostock Germany; ^3^ Department of Biomedical Sciences University of Antwerp Antwerp Belgium; ^4^ Microglia and Inflammation in Neurological Disorders (MIND) Lab VIB‐Center for Molecular Neurology (CMN), VIB Antwerp Belgium; ^5^ Department of Neurology Rostock University Medical Center Rostock Germany; ^6^ Institute of Clinical Chemistry and Laboratory Medicine Rostock University Medical Center Rostock Germany; ^7^ Center for Transdisciplinary Neurosciences Rostock (CTNR) Rostock University Medical Center Rostock Germany; ^8^ German Center for Neurodegenerative Diseases (DZNE) Research Site Rostock/Greifswald Rostock Germany

**Keywords:** aging, aging clock, amyotrophic lateral sclerosis, FUS, microglia, nucleocytoplasmic shuttling, progerin, senolytics

## Abstract

Microglia, the brain's resident immune cells, are crucial for maintaining healthy brain homeostasis. However, as the brain ages, microglia can shift from a neuroprotective to a neurotoxic phenotype, contributing to chronic inflammation and promoting neurodegenerative processes. Despite the importance of understanding microglial aging, there are currently few human in vitro models to study these processes. To address this gap, we have developed a model in which human microglia undergo accelerated aging through inducible progerin expression. HMC3‐Progerin cells display key age‐related markers such as activation of the senescence‐associated secretory phenotype (SASP) as well as an increase in DNA damage. These prematurely aged HMC3 cells show a reduced response to LPS activation, exhibit impairments in essential microglial functions including decreased migration and phagocytosis as well as transcriptomic alterations including a shift observed in aging and neurodegeneration. Additionally, we observed an impaired stress response and a defect in nucleocytoplasmic transport, especially affecting the amyotrophic lateral sclerosis (ALS) associated protein FUS. This suggests that microglia play a contributory role in driving neurodegenerative processes in the aging brain. Our microglia aging model offers a valuable tool for exploring how aged microglia affect brain function, enhancing our understanding of their role in brain aging.

## Graphical abstract

We developed a human microglia aging model by doxycycline‐induced GFP‐progerin expression, which offers a valuable tool to study human microglial aging and its contribution to neurodegeneration. Induced HMC3‐Progerin cells exhibited increased age markers as well as transcriptomic alterations, including an age‐associated transcriptomic shift and overlapping signatures observed in aging and neurodegeneration, as well as functional alterations (e.g., in stress response, defect in nucleocytoplasmic transport). Senolytic treatments partially reversed some age markers.

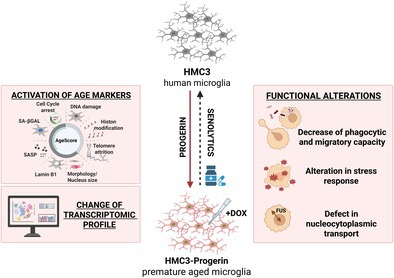

## 1 Introduction

Brain aging is one of the most significant risk factors for developing neurodegenerative diseases such as Amyotrophic lateral sclerosis (ALS) or Alzheimer's disease (AD). The aging process is described as a time‐dependent functional decline (López‐Otín et al. 2013) and includes processes that reduce the health and survival of individuals, organs, or cells (Hartmann et al. 2023). As brain aging can trigger neurodegenerative processes, it is important to study these in more detail.

Microglia, the brain's primary immune cells, play a crucial role in maintaining brain homeostasis. They originate early in embryonic development within the yolk sac before migrating into the brain (Wolf et al. 2017). Once in their final location, microglia strongly reduce their cell soma and form distinct branches with which they continuously monitor their environment, referred to as “homeostatic” microglia. Throughout development and life, microglia contribute to brain homeostasis by eliminating unnecessary synapses through a process known as synaptic pruning (Wolf et al. 2017). Upon activation by external stimuli, such as apoptotic cells or abnormal protein aggregates, microglia retract their processes and acquire the capacity for migration and phagocytosis (Wolf et al. 2017). This dynamic regulation between activated and homeostatic microglia is essential for maintaining brain health. However, this balance becomes increasingly disturbed with advancing age.

The physiological aging of microglia was investigated in human post‐mortem tissue already in 2004 (Streit et al. 2004). It was observed that microglia adopt a dystrophic morphology with increasing age, characterized by deramification and shortening of processes as well as cytoplasmic fragmentation. As a result, their ability to surveil the brain declines significantly with age (Antignano et al. 2023). Moreover, aged microglia become hyperactivated, leading to increased secretion of inflammatory cytokines and altered phagocytic function (Antignano et al. 2023). This shift in microglial phenotype contributes to impaired synaptic networks and diminished recovery following injury. Since neurons heavily depend on glial cells—particularly microglia—for survival, function, and protein turnover, the aging of microglia has a profound impact on neuronal viability (Antignano et al. 2023). In contrast to the youthful brain, microglia in the aged brain display reduced migration but enduring inflammation upon injury (Damani et al. 2011; Hefendehl et al. 2014) in line with studies reporting reduced phagocytosis and increased ROS production favoring an inflammatory environment (Koellhoffer et al. 2017). Other reported properties in aged microglia are increased DNA damage and telomere shortening (von Bernhardi et al. 2015). Furthermore, several studies display a change from a neuroprotective anti‐inflammatory to a neurotoxic pro‐inflammatory phenotype with old age (Galatro et al. 2017; Olah et al. 2018) that support the dysfunction of the innate and adaptive immune system associated with aging. Similarly, during the course of neurodegenerative diseases, homeostatic microglia adapt their phenotype from a neuroprotective, anti‐inflammatory to a disease‐associated, pro‐inflammatory phenotype (diseased associated microglia [DAMs]), which may fundamentally contribute to the progression of these diseases (Chiu et al. 2013; Krasemann et al. 2017). One of the first phenotype switches to DAMs was described in SOD1 mice, the most commonly used mouse model for ALS (Chiu et al. 2013).

ALS is a neurodegenerative disease characterized by the selective loss of motor neurons. Commonly mutated genes include *FUS* (Fused in sarcoma), *SOD1* (superoxide dismutase 1) and *TARDBP* (TAR DNA binding protein, TDP‐43 protein). TDP‐43 and FUS are RNA‐ and DNA‐binding proteins that are predominantly localized in the nucleus but are capable of nucleocytoplasmic shuttling (Kino et al. 2011). FUS plays a critical role in DNA repair and RNA metabolism (Kino et al. 2011). Most ALS‐associated *FUS* mutations disrupt its nuclear import, leading to cytoplasmic mislocalization due to mutations within or near the C‐terminal nuclear localization sequence (NLS) (Vance et al. 2013). In recent years, the pathological role of microglia in ALS has gained increasing attention. For instance, pro‐inflammatory cytokine production was elevated in primary microglial cultures after exposure to conditioned medium from astrocytes overexpressing wild‐type FUS (Ajmone‐cat et al. 2019). Mice overexpressing wild‐type FUS also exhibit microglial activation in the spinal cord (Mitchell et al. 2013). Furthermore, in mice expressing a truncated FUS protein without a nuclear localization signal, pro‐ and anti‐inflammatory microglial activation was observed during the symptomatic phase (Funikov et al. 2018). Despite this growing recognition of microglia's involvement in ALS, the specific role of aged microglia in neurodegenerative processes remains unexplored. This knowledge gap is likely due to the absence of suitable model systems to study these interactions in depth.

Although it is well‐established that murine and human microglia age differently (Antignano et al. 2023), the majority of studies on microglial aging have been conducted in rodent models. In aged mice, typical brain changes include reduced cortical thickness, enlarged ventricles, cerebral microbleeds, and lipofuscin accumulation (Taylor et al. 2020). Accelerated aging in mouse models has been induced through DNA repair deficiencies (Ercc1^−/−^) or telomere shortening (mTerc^−/−^) (McWhir et al. 1993; Raj et al. 2015), both exhibiting systematic aging characteristics. The Ercc1^−/−^ mice suffer from kyphosis and a reduced life span (McWhir et al. 1993). In constitutive Ercc1^−/−^ mice, microglia show increased phagocytosis, proliferation, and heightened responses to LPS‐induced inflammation. In contrast, microglia‐specific Ercc1 deletion neither triggered activation nor increased LPS reactivity. Gene expression analysis suggests a transient aging signature, distinct from primed or DAM profiles (Zhang et al. 2021). The mTerc^−/−^ mouse model lacks telomerase activity, and microglia from this model display an enhanced answer of pro‐inflammatory genes after a peripheral LPS injection. However, this heightened response was not associated with genes linked to aged microglia and correlated closely with immune cell infiltration (Raj et al. 2015). In these existing models, only whole brain aging was studied, and therefore the specific aging effects originating from microglia themselves were not addressed.

Human models of microglial aging remain limited. While primary microglia can be isolated from human postmortem brain tissue using magnetic cell sorting (MACS) or fluorescent activated cell sorting (FACS), yields are low and isolation may induce artificial or activated phenotypes (Aktories et al. 2022). Microglia‐like cells can also be differentiated from induced pluripotent stem cells (iPSC) (Haenseler et al. 2017). However, rejuvenation takes place during de‐differentiation to the stem cell stage, whereby most of the age markers get lost (Tang et al. 2017).

We thus developed a model for accelerated aging in HMC3 cells (human microglial clone 3 cell line) by utilizing doxycycline‐inducible expression of progerin, a mutated form of Lamin A responsible for Hutchinson‐Gilford progeria syndrome (HGPS). Patients suffering from this hereditary progeria syndrome display accelerated aging (Scaffidi and Misteli 2006), making it a reasonable cell culture model to study aging processes (del Campo et al. 2019). The HMC3 cell line was established through SV40‐dependent immortalization of a human fetal brain‐derived primary microglia culture (Dello Russo et al. 2018). Using our previously published AgeScore (Hartmann et al. 2023), we characterized HMC3‐Progerin cells and demonstrated a significant increase in various age markers. Functionally, prematurely aged microglia displayed a decline in migration and phagocytosis as well as transcriptomic changes. Furthermore, induced HMC3‐Progerin cells displayed mechanistic defects that reconfirm the important role of microglia in the development of neurodegenerative processes. Finally, several of the age‐associated changes could be restored using senolytics putatively already available for repurposing strategies.

## 2 Material and Methods

### 2.1 Cell Culture

Human HMC3 cells (ATCC, CRL‐3304) were cultured in MEM α medium (Gibco, #12571063) supplemented with 10% fetal bovine serum and 1% penicillin–streptomycin. Microglia were split using 0.05% trypsin‐EDTA for 5 min. Culture medium was changed every 2–3 days. To induce progerin expression, cells were treated with 1 μg/mL doxycycline (Merck, #D3072) for 72 h. To examine cell growth and cell death in induced (+DOX) in comparison to non‐induced (‐DOX) HMC3‐Progerin, 2 × 10^5^ cells were seeded in a 6‐well plate in MEM α medium without or with 1 μg/mL doxycycline. The number of live and dead cells was assessed and quantified using Trypan blue (Sigma‐Aldrich, #T8154, dilutes 1:1) with a CellDrop FL (DeNovix) at 24, 48, and 72 h post‐seeding. For activation of microglia, cells were treated with 10 μg/mL Lipopolysaccharide (LPS, Merck, # L2630) for 24 h. For treatment with senolytics, cells were treated at each medium change with either 500 nM rapamycin or a combined treatment of 200 nM dasatinib and 10 μM of quercetin for 3, 7, or 14 days. For stress granule (SG) induction, cells were treated with 200 μM sodium arsenite (SA) for 1 h.

### 2.2 Generation of Inducible Progerin Expression in HMC3

HMC3 cells were transduced with lenti‐associated viruses (LAV) containing (1) GFP‐Progerin and (2) rtTA3. In detail, LAVs were generated by seeding 8 × 10^6^ HEK293t cells on a 10 cm petridish and transfecting them the next day using Lipofectamin2000 (Invitrogen, #11668027) with 1800 ng of psPAX2 (gift from Didier Trono; Addgene plasmid #12260), 200 ng VSV‐G (gift from Akitsu Hotta; Addgene plasmid #138479) and 2000 ng of pLenti‐CMV‐TREG3‐Neo‐GFP‐Progerin (gift from Tom Misteli; Addgene plasmid #118710) or pLenti‐CMV‐rtTA3‐Hygro (gift from Eric Campeau; Addgene plasmid #26730). Medium was changed 18 h later, and LAVs were harvested 48 and 72 h after transfection by centrifugation (1250 rpm, 5 min) and filtration (45 μm) of the medium. One week after transduction of HMC3 with the above‐mentioned LAVs (MOI of 2) together with 6 μg/mL Polybrene (Merck, #TR‐1003‐G), antibiotic selection was done with 300 μg/mL Hygromycin B (InvivoGen, #ant‐hg‐1) and 750 μg/mL G418 (InvivoGen, #ant‐gn‐1) for two weeks. Due to this selection, HMC3‐Progerin cells most likely belong to the same parental clone. For the experiments reported, we used HMC3‐Progerin cells between passages 10 and 30 after selection.

### 2.3 Western Blotting

Cells were lysed in RIPA Buffer (50 mM Tris pH 7.2, 150 mM NaCl, 0.1% SDS, 0.5% sodium deoxycholate, 1% Triton X100, 1% EDTA [0.5 M], 1% NP40) containing protease inhibitor cocktail (Roche, #04693116001). Protein concentration of lysates was determined by BCA assay (Thermo Scientific, #23225). Lysate was mixed with Laemmli buffer, incubated at 95°C for 4 min, and loaded onto a 10% Bis/Tris SDS gel. Proteins were detected with different antibodies (Table S1) in a Li‐COR Odyssey XF Dual Mode imaging system. Quantification was done with Empiria Studio v2.2.

### 2.4 Immunofluorescence Staining (IF)

HMC3‐Progerin cells were seeded at a density of 3.5 × 10^3^ per well for 3‐day experiments, 1.75 × 10^3^ per well for 7‐day experiments, and 0.875 × 10^3^ per well for 14‐day experiments in an 18‐well slide (Cellvis, #C18‐1.5H) and incubated for 3, 7, or 14 days with or without 1 μg/mL doxycycline. Cells were fixed with 4% PFA at 37°C for 20 min followed by permeabilization with 0.2% of TritonX for 10 min at RT and blocking with Pierce Protein‐Free T20 TBS Blocking Buffer (ThermoFisher Scientific, #37571) at RT for 1 h. Primary antibodies (Table S2) were incubated overnight at 4°C. After washing, secondary antibodies (Table S2) were incubated for 90 min. Finally, after repeated washing, cells were mounted with 4.6‐diamidino‐2‐phenylindole (DAPI) Fluoromount‐G (Southern Biotech, #0100–20) and images were taken with a LSM900 confocal microscope (Zeiss). Analysis was done with Fiji software (v1.53c). γH2A.X foci were counted per nucleus (threshold: 20–200; size: 5). Corrected total cell fluorescence (CTCF) was calculated as following: (CTCF) = Integrated Density—(Area of selected cell/region × fluorescence of background readings). For each image, we selected a region of interest (ROI) surrounding the nucleus based on DAPI staining. The background fluorescence was measured in an adjacent cell‐free area of the same image and at a similar size and intensity range. The same background ROI was used to correct all cells within the image. Nucleus size was determined with area (pixel units) of nucleus with Fiji software. Further analysis of the nucleus morphology was done in accordance with Janssen et al. (2022). The ratio of nucleus/cytoplasmic (nuc/cyt) proteins was calculated with CellProfiler (v4.2.6). For each quantification, more than 20 cells were analyzed per biological replicate.

### 2.5 Determination of RNA Expression Levels

Total RNA was isolated using the NucleoSpin RNA purification kit (Machery‐Nagel, #740955.250) according to the manufacturer's protocol, and cDNA was generated from 250 ng isolated RNA with the High‐Capacity cDNA Reverse Transcription Kit (Thermo Fisher Scientific, #4368814). mRNA expression levels were determined using Rotor Gene (QIAGEN) with QuantiNova SYBR Green PCR Kit (QIAGEN, #208056) using the primers summed up in Table S3. qPCRs were performed in duplicates for each sample (*n* = 1 correspond to 2 technical replicates). Data sets were normalized relative to GAPDH or 18S using the delta–delta‐CT method. Data sets are presented as relative expression in comparison to GAPDH or 18S as mean with SD.

### 2.6 Telomere Length Measurement via Monochromal Multiplex qPCR (MM‐qPCR)

A standard extraction kit (DNeasy Blood and Tissue Kit, Qiagen, Cat. #69504) was used for DNA extraction. Mean telomere length was determined using the modified MM‐qPCR as described previously (Hartmann et al. 2023). DNA samples (20 ng/μL) and a reference DNA standard (0.137–100 ng/μL) were assayed in triplicates on different plates, and the average of three measurements was used to report the mean telomere length for each sample. A non‐template control (water) and a positive control (human leukemia cell line 1301 DNA) were prepared in duplicates and run on every plate. The standard includes DNA samples of 352 healthy donors, with an average age of 40.14 years (18–70 years old; 38.35% males and 61.65% females). The assay was performed using a BioRad CFX384 real‐time C1000 thermal cycler with the following profile: 1 cycle of 15 min at 95°C; 2 cycles of 15 s at 94°C, 1 cycle of 15 s at 49°C; 40 cycles of 15 s at 94°C, 1 cycle of 10 s at 62°C, 1 cycle of 15 s at 72°C with T signal acquisition, 10 s at 85°C, and 15 s at 89°C with signal acquisition. PCR reagents were used at the following final concentrations: 1 U titanium Taq DNA polymerase per reaction with provided titanium Taq PCR buffer (Takara, Cat. # 639208), SYBR Green I (Invitrogen, #S7563), 0.2 mM of each dNTP, 1 mM DTT, 1 M betaine, 900 nM of each telomere primer (Telg, Telc, Table S3) and 300 nM of each single‐copy gene primer (ALBu, ALBd, Table S3). The ratio of telomere to single‐copy gene content (TLR) is taken as a relative measurement of telomere length and expressed in arbitrary units. The intra‐assay coefficients of variation were < 0.3 for all samples.

### 2.7 Senescence‐Associated β‐Galactosidase (SA‐β‐GAL‐Assay)

Activity of SA‐β‐Gal was determined using the Cellular Senescence Detection Kit—SPiDER Blue (Dojindo, #SG07‐10) according to the manufacturer's protocol. Images were taken with a LSM900 confocal microscope (Zeiss) and analysis was done with Fiji software by measuring the mean fluorescence intensity.

### 2.8 Enzyme‐Linked Immunosorbent Assay (ELISA)

ELISA from cell culture supernatants was performed using the Human IL‐6/Interleukin‐6 ELISA Kit PicoKine (BosterBio, #EK0410) or the Human IL‐8/Interleukin‐8 ELISA Kit PicoKine (BosterBio, #EK0413) according to the manufacturer's recommended procedures. ELISA was performed in duplicates for each sample (*n* = 1 correspond to the mean of 2 technical replicates).

### 2.9 Cell Cycle Analysis

HMC3‐Progerin cells were seeded in a 6‐well plate and incubated at 37°C and 5% CO_2_. After 24 h, cells were treated with 7.5 μg/mL of Mytomycin C (Merck, #10107409001) which was removed by a complete medium change after 2 h. After further incubation for 24 or 48 h, cells were detached using Trypsin, washed once, and resuspended in 500 μL MACS Separation Buffer (Miltenyi Biotec #130‐091‐221). Cell density and vitality were determined using Trypan Blue and CellDrop FL (DeNovix). Fixation was performed by using ice‐cold 70% ethanol, and samples were stored at 4°C for up to one week. For analysis, cells were washed once and stained with FxCycleTM PI/RNase Staining Solution (Invitrogen, #F10797) according to manufacturer instructions. Flow cytometry was conducted using a FACSCalibur cytometer with a 488 nm laser and CellQuest Pro software (both Becton Dickinson). Data analysis was performed using FlowJo 10.08.1. Here, single cells were gated using a forward‐ and side‐scatter plot, and cell cycle phases were determined within the PI histogram by gating the first peak (G0/G1 phase), the second peak (G2/M phase) and the cells in between (S phase) with the same gates for all samples of a given time point.

### 2.10 Migration Assay

Overall migration of HMC3‐Progerin cells was assayed with 2‐well‐migration inserts (Culture‐Insert 2 Well in μm dish 35 mm, ibidi, #81176). 3.5 × 10^4^ HMC3‐Progerin cells were seeded into each insert‐chambers in 70 μL MEM α media (Gibco, #12571063) without/with doxycycline supplemented with 10% fetal bovine serum and 1% penicillin–streptomycin. After 24 h, 7.5 μg/mL Mitomycin C (Merck, #M5353) to inhibit cell proliferation was added for 2 h to the cells. Afterwards, 2 mL of PBS was added to the dish and inserts were removed carefully. Cells were washed once with 1 mL of PBS. After washing, 2 mL of medium was added. Longitudinal assessment of migration was performed every day by taking 6 representative pictures at defined positions with an Axiovert 40 CFL microscope (Zeiss) until the migrating cells covered the scratch. Quantification was done with Fiji plugin “MRI wound healing tool” with the first and the last day of assay documentation.

### 2.11 Phagocytosis Assay

For investigating phagocytosis of HMC3‐Progerin cells, 2 × 10^4^ cells per well were seeded in an 8‐well μ‐Slide (ibidi, #80806) and incubated for 72 h with or without 1 μg/mL doxycycline. On the day of the experiment, cell culture medium was replaced and pHrodo Red Zymosan BioParticles (Thermo Fisher Scientific, #P35364) diluted 1:100 in cell culture medium was added to the cells for 3 h. Cells were fixated and stained as described before. The cytoplasm around each nucleus was identified via P2RY12R (Table S2) IF‐staining and pHrodo red‐positive spots within this region were detected. For quantification, the total amount of phagocytosing cells was counted.

### 2.12 RNA Sequencing

For RNA sequencing, 1 × 10^6^ non‐induced and induced HMC3‐Progerin cells were collected. RNA extraction and library preparation with PolyA amplification was performed at Genewiz. A second sequencing was done at Novogene to increase sample number. Libraries were sequenced as 150 bp on fragments with an average of 28 million (ranging from 22 – 39 M) read pairs on the Illumina NovaSeq. Using DESeq2, a comparison of gene expression between HMC3‐Progerin cells with/without doxycycline treatment was performed. The Wald test was used to generate *p*‐values and log2 fold changes. Genes with an adjusted *p*‐value < 0.05 and absolute log2 fold change > 1 were defined as differentially expressed genes (DEGs). This RNAseq Illumina sequencing data is available at the European Nucleotide Archive, under the project accession number PRJEB81801.

### 2.13 Shuttling Assay

To examine the function of nucleocytoplasmic transport (NCT), HMC3‐Progerin cells were seeded at a density of 2 × 10^4^ per well in an 8‐well μ‐Slide (ibidi, #80806). Afterwards, cells were transduced using 2 MOI of lentiviral NLS‐NES‐tagged tdTomato (gift from Jeffrey Rothstein; Addgene plasmid #112579, NLS‐NES‐tagged tdTomato) and 6 μg/mL Polybrene (Merck, #TR‐1003‐G). After incubation over 72 h, cells were fixed and stained as described before. Quantification was done by measuring the ratio of fluorescence intensity as well as the mean fluorescence intensity of the nucleus and cytoplasm via CellProfiler (v4.2.6).

### 2.14 Statistics

Multiple biological replicates (independent cell culture batches under identical conditions) were used for all experiments. Statistical analyses were performed using GraphPad Prism 8 (LaJolla). Experimental groups were compared using student's *t*‐test, one‐way ANOVA, or two‐way ANOVA (followed by Tukey or Sidak multiple comparison test). Statistical significance was set at *p*‐values < 0.05 (*), < 0.01 (**), < 0.001 (***). Data were plotted using GraphPad Prism 8 (LaJolla) showing mean with SD. Unless stated otherwise, *n* refers to independent biological replicates (separate experiments using independently cultured and treated cells). For imaging, multiple non‐overlapping images per well were acquired, but statistical analysis was based on the per‐well average, with each well representing one biological replicate. Technical replicates ensured consistency but were not included in statistical testing.

## 3 Results

### 3.1 Prematurely Aged HMC3‐Progerin Cells Exhibit Activation of Various Age Markers and an Elevated AgeScore

**FIGURE 1:**
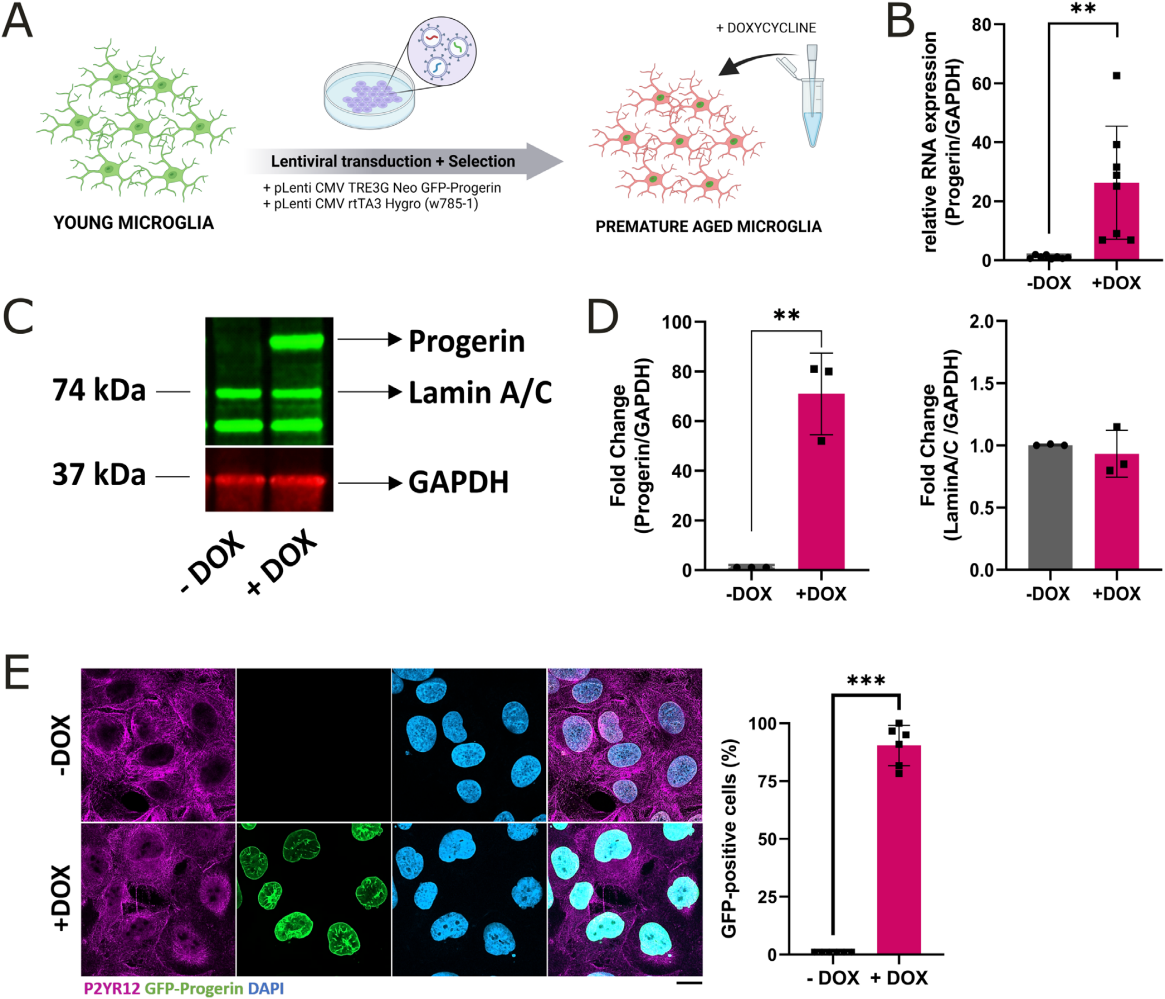
Overexpression of progerin to induce premature aging in the immortalized microglial cell line HMC3. (A) Schematic overview of lentiviral transduction strategy using pLenti‐CMV‐TRE3G‐Neo‐GFP‐Progerin and pLenti‐CMV‐rtTA3‐Hygro constructs, resulting in a doxycycline inducible microglial cell line. (B) QPCR analysis revealed a significant upregulation of progerin mRNA in HMC3‐Progerin cells after 72 h of doxycycline treatment (+DOX) compared to non‐induced controls (−DOX) [*n* = 8, mean ± SD, unpaired students *t*‐test]. (C, D) Western Blot analysis confirmed a corresponding increase in progerin protein levels in doxycycline‐treated cells (+DOX) relative to untreated controls (−DOX) after 72 h [*n* = 3, mean ± SD, unpaired students *t*‐test]. (E) Representative pictures of GFP‐progerin expression in induced HMC3‐Progerin cells. Images show robust GFP‐progerin expression in induced cells. Quantification indicated a transduction efficiency of 89.42% after 72 h of doxycycline induction and selection [scale bar = 20 μm; *n* = 6 (each *n* tested > 20 cells), mean ± SD, unpaired student's *t*‐test].

We first established a human model to investigate microglial aging using an inducible progerin expression TET‐ON system that was recently established for drug screening in progeria syndrome using patient‐derived fibroblasts (Hartmann et al. 2023; Kubben et al. 2016). HMC3 cells were transduced with two different LAVs carrying either the *GFP‐Progerin* or the *rtTA3* gene, followed by an antibiotic selection with G418 and Hygromycin B (Figure 1A). Once stable cell lines were established, progerin expression was induced by treating the cells with 1 μg/mL doxycycline for 3 days. Induced HMC3‐Progerin cells (+DOX) displayed a significant increase in RNA (Figure 1B) as well as in the protein expression level of progerin (Figure 1C,D) whereas levels of endogenous Lamin A/C expression were not changed. The transduction efficiency after 3 days of doxycycline induction was 89.42% ± 3.5 (Figure 1E). Doxycycline treatment itself had no impact on cell growth (Figure S1A) and did not induce cell death or DNA damage (Figure S1B,C).

**FIGURE 2:**
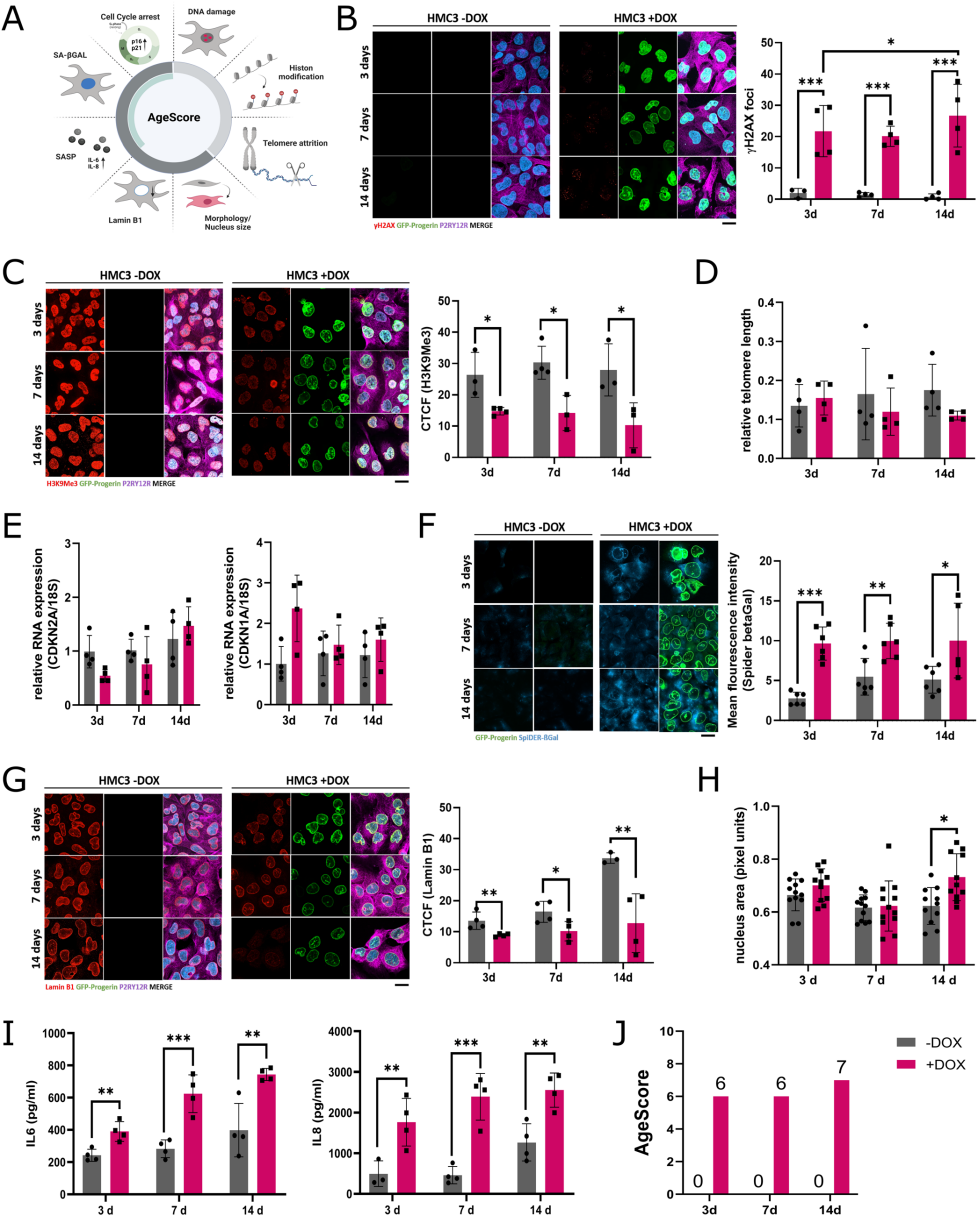
Determination of the AgeScore in prematurely aged HMC3‐Progerin cells. (A) Schematic overview of the AgeScore that includes: Age markers reflecting the primary (DNA damage, histone modification, telomere attrition) and antagonistic (cell cycle arrest, senescence‐associated β‐Galactosidase (SA‐βGAL), senescence‐associated secretory phenotype (SASP), Lamin B1 expression, morphology) hallmarks of aging that include typical markers of cellular senescence (green bar). (B) Representative images and quantification of γH2A.X foci after 3, 7 and 14 days of doxycycline treatment show increased DNA damage in induced (+DOX) HMC3‐Progerin cells compared to non‐induced (−DOX) controls, with progressive accumulation over time. (C) H3K9 trimethylation (H3K9Me3) levels were significantly reduced in induced (+DOX) HMC3‐Progerin cells at all time points, as determined by corrected total cell fluorescence (CTCF) analysis. (D) Telomere length, assessed by monochrome multiplex quantitative PCR (MM‐qPCR), remained unchanged relative to a standard derived from healthy mixed‐aged individuals. (E) mRNA expression of cell cycle regulators *CDKN2A* (p16) and *CDKN1A* (p21) showed no significant induction following progerin expression, as measured by qRT‐PCR. (F) SA‐βGal activity, assessed by Spider βGal staining, was significantly increased in induced (+DOX) HMC3‐Progerin cells at all time points. For each n we tested > 100 cells. G: Representative images and CTCF quantification revealed a marked reduction in Lamin B1 expression after 3, 7 and 14 days of progerin induction. (H) Quantification of nuclear area (in pixel units) showed a significant increase only after 14 days of doxycycline treatment. (I) Enzyme‐linked immunosorbent assay (ELISA) of conditioned medium indicated elevated secretion of interleukin 6 (IL6) and interleukin 8 (IL8) in induced (+DOX) HMC3‐Progerin cells. (J) Calculation of AgeScore demonstrate an increase from 0 to 6 (after 3 and 7 days) and an increase from 0 to 7 after 14 days of doxycycline treatment in progerin expressing HMC3 microglia. *All data are presented as mean ± SD; statistical significance was determined by two‐way ANOVA followed by Sidak's post hoc test (*p < 0.05, **p < 0.001, **p < 0.0001); scale bar = 20 μm.

Next, we calculated the effects of progerin induction on the AgeScore, which we recently developed to provide a quantitative and comparative tool to measure aging‐associated cellular responses under defined conditions (Hartmann et al. 2023). The AgeScore comprises a panel of classic age markers, measurable characteristics used to estimate biological age or related conditions, thereby capturing various aging processes within a single score (Figure 2A). This allows us to estimate the biological age of in vitro cell culture systems. To assess the effect of prolonged progerin expression, cells were treated with doxycycline for 3, 7 and 14 days.

First, we examined the activation of markers reflecting the primary hallmarks of aging (heterochromatin loss, DNA damage, telomere attrition) in induced HMC3‐Progerin cells. Primary hallmarks of aging are considered to be the main causes of cellular damage associated with rising age (López‐Otín et al. 2013). To assess DNA damage, we quantified the number of DNA double‐strand breaks (DSBs) by staining of γH2A.X foci. We observed a significant increase in the number of DSBs in microglia expressing progerin at all three time points (Figure 2B). DNA damage increases significantly between 3 and 14 days of treatment. Furthermore, induced HMC3‐Progerin displayed a decrease in the expression level of H3K9Me3 at all three time points measured by the corrected total cell fluorescence (CTCF) compared to the non‐induced control cells (Figure 2C). Telomere length measured by monochromatic multiplex qPCR (MM‐qPCR) was not changed (Figure 2D).

Furthermore, we investigated markers reflecting the antagonistic hallmarks of aging (cell cycle arrest, activation of SA‐βGal expression, activation of the senescent‐associated secretory phenotype (SASP), Lamin B1 expression, morphological changes), reactions that initially are intended to mitigate the damage but if chronically present become harmful themselves (López‐Otín et al. 2013). Lamin B1 is considered an antagonistic age marker because its decline is a hallmark of cellular senescence. Although its expression decreases with age, this reflects a stress response that initially preserves cellular integrity but becomes detrimental over time, contributing to nuclear abnormalities and chromatin disorganization (Freund et al. 2012). Induced HMC3‐Progerin cells showed no changes in RNA expression levels of *CDKN2A* (p16) and *CDKN1A* (p21), two key markers of cell cycle arrest (Figure 2E). We additionally determined the protein expression levels of p16, p21, and KI67 using IF stainings (Figure S1). We were unable to detect any changes here, either. However, SA‐βGAL expression increases in induced HMC‐Progerin cells (Figure 2F). Additionally, they displayed reduced Lamin B1 expression at all three time points (Figure 2G), while changes in nucleus size were only observed after 14 days of progerin expression (Figure 2H). To gain deeper insight into nuclear changes, we quantified both solidity and form factor (Figure S2D–G). The solidity is the ratio of the area of the nucleus to the area of its convex hull, whereby the form factor is a measure of how close the shape of the nucleus is to a perfect circle (Janssen et al. 2022). We observed a decrease in the form factor, whereas nuclear solidity remained unchanged. This indicates that progerin expression induces subtle but specific changes in nuclear morphology, leading to a less circular and more irregular nuclear shape without affecting overall nuclear compactness. SASP activation was assessed by measuring IL6 and IL8 secretion into the cell culture medium, revealing a significant increase in both cytokines in induced HMC3‐Progerin compared to non‐induced control cells (Figure 2I). Overall, the AgeScore increases from zero in the microglia without progerin expression to a value of six after 3 and 7 days and to a value of seven after 14 days in induced HMC3‐Progerin cells (Figure 2J, Table 1). Since this index is a categorical sum based on the statistical quantification of age marker measurements and not a continuous measurement at the replication level, SD is not shown for AgeScore plots.

**TABLE 1:** Calculation of the AgeScore of prematurely aged HMC3‐Progerin cells.

Age marker category	Age marker	Tested via	HMC3‐Progerin‐DOX	HMC3‐Progerin + DOX		
				3 days	7 days	14 days
Primary age markers	DNA‐damage	γ‐H2AX	0	1	1	1
	Histone modification	H3K9Me3	0	1	1	1
Primary AgeScore			**0**	**2**	**2**	**2**
Antagonistic age marker	Cell cycle arrest	p21	0	0	0	0
		p16	0	0	0	0
	SA‐β‐GAL activity	SA‐β‐Gal assay	0	1	1	1
	SASP	IL‐6	0	1	1	1
		IL‐8	0	1	1	1
	Change in lamina	Lamin B1	0	1	1	1
	Morphology	Nucleus area	0	0	0	1
Antagonistic AgeScore			**0**	**4**	**4**	**5**
Full AgeScore			**0**	**6**	**6**	**7**

*Note:* To create this score cells were rated as “1” for a significant (positive) age marker result and as “0” for each non‐significant (negative) result. Subsequently, results of tested age markers were summed up to calculate the primary and the antagonistic AgeScore. Finally, both scores together resulted in the full AgeScore (Hartmann et al. 2023).

### 3.2 Premature Aging of HMC3 Microglia Leads to a Decline in Microglial Function

**FIGURE 3:**
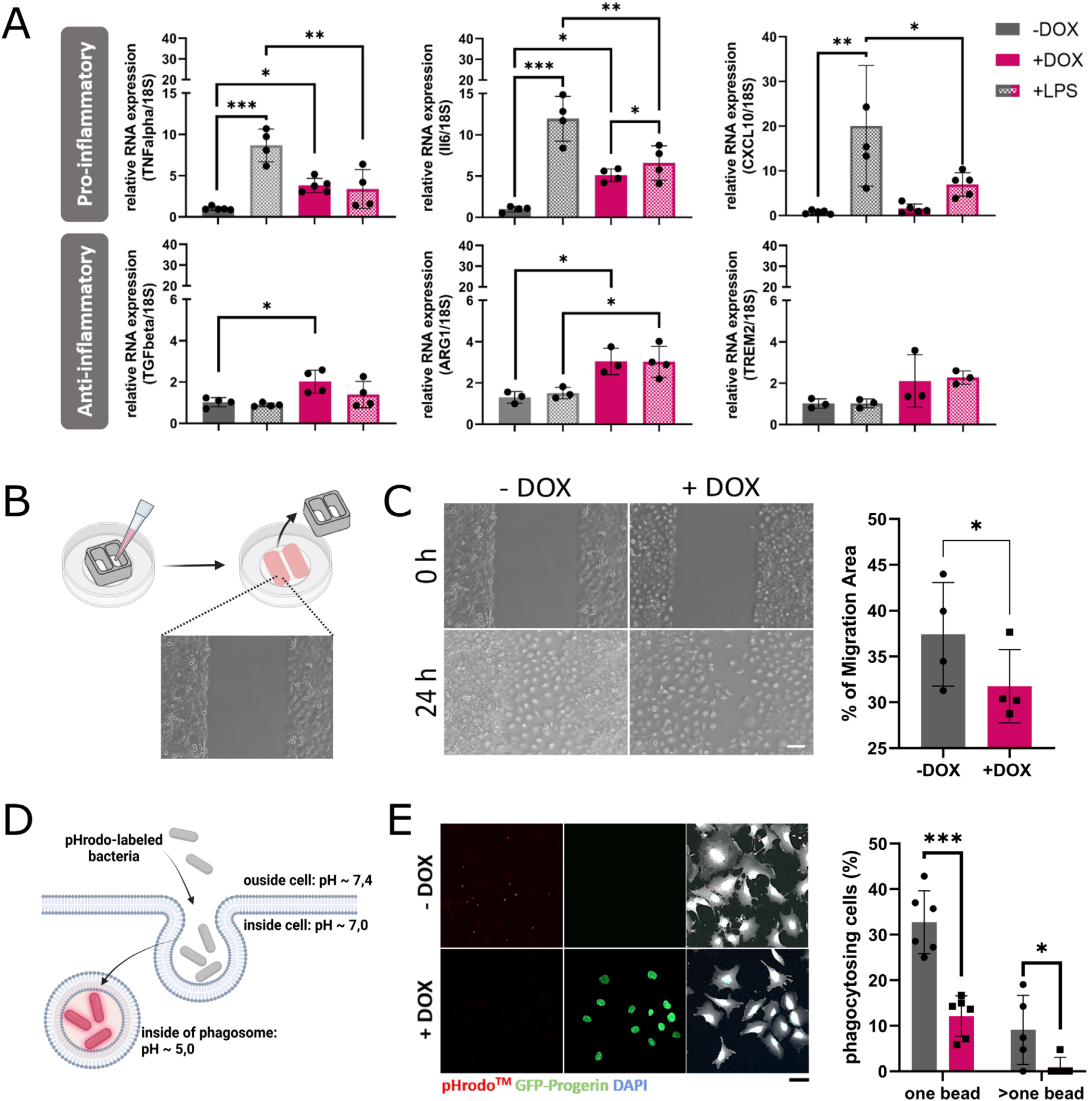
Functional decline of prematurely aged HMC3‐Progerin cells. (A) RNA expression analysis of pro‐ and anti‐inflammatory genes by qRT‐PCR after 72 h of doxycycline‐induced aging and 24 h of stimulation with 10 μg/mL lipopolysaccharide (LPS). Prematurely aged HMC3‐Progerin cells (+DOX) showed increased expression of pro‐inflammatory markers compared to non‐induced controls (−DOX). (B) Schematic overview of the migration assay using 2‐well culture inserts to generate a defined cell‐free gap. (C) Representative images and quantification of microglial migration 24 h after insert removal revealed significantly reduced migration capacity in doxycycline‐induced (+DOX) HMC3‐Progerin compared to non‐induced (−DOX) control cells. Cells were pretreated with 7.5 μg/mL mitomycin C to inhibit proliferation (see Figure S3A). (D) Schematic overview of phagocytosis assay using pHrodo Red Zymosan BioParticles, which fluoresce under acidic conditions in phagosomes. (E) Representative images and quantification demonstrated significantly reduced phagocytic activity in doxycycline‐induced (+DOX) HMC3‐Progerin cells compared to non‐induced (−DOX) controls. *All data are shown as mean ± SD; statistical significance was determined using unpaired student's *t*‐test (C, E) or one‐way ANOVA with Tukey's post hoc test (A); *p < 0.05, **p < 0.001, **p < 0.0001; scale bars = 50 μm.

Microglia are the brain's first line of defense. Therefore, their central functions are to migrate towards locations of an infection or destruction and to phagocytose bacteria, debris, or apoptotic cells after activation. As it is known that microglia with upcoming age are increasingly hyperactivated and lose their migration and phagocytosis capacity, we wanted to examine these important functions upon induced premature aging. Therefore, we analyzed the RNA expression levels of various pro‐inflammatory (*TNFalpha* [tumor necrosis factor alpha], *IL6* [interleukin 6], *CXCL10* [CXC motif chemokine ligand 10]) and anti‐inflammatory (*TGFbeta* [transforming growth factor beta], *ARG1* [arginase 1], *TREM2* [triggering receptor expressed on myeloid cells 2]) factors after inducing premature aging by doxycycline treatment for 3 days (Figure 3A). To further assess microglial activation, we treated the cells with 10 μg/mL LPS for 24 h. In induced HMC3‐Progerin cells, we observed an increase in the basal expression of the pro‐inflammatory genes *TNFalpha* and *IL6* as well as an upregulation of anti‐inflammatory genes *TGFbeta* and *ARG1*. A significant upregulation in premature aged HMC3‐Progerin cells was only observed for IL6.

The migratory capacity of microglia was assessed by scratch assay using 2‐well inserts (Figure 3B). To specifically evaluate the migration of HMC3‐Progerin cells, proliferation was inhibited by treatment with Mitomycin C for 2 h before insert removal. As described for other cell lines, Mitomycin C treatment prevented cell cycle progression by slowing down the S phase exit and by accumulation of cells in the G2/M phase (Figure S3A–C). Increased cell death could not be detected (Figure S3D). After insert removal, cells were monitored longitudinally over 48 h. We observed a significant reduction in the migration of induced HMC3‐Progerin compared to non‐induced cells (Figure 3C).

Phagocytic activity was analyzed using pHrodo Red Zymosan BioParticles, which fluoresce at a pH below 5, typically found inside phagosomes (Figure 3D). After incubation with pHrodo particles, the percentage of phagocytic cells was quantified. We found a significant decline in the phagocytic capacity in induced compared to non‐induced HMC3‐Progerin cells (Figure 3E). This decrease was observed when counting both cells with at least one bead and those with multiple beads. A decline in the phagocytosis capacity was also seen after 7 and 14 days of progerin expression (Figure S3E,F). However, P2RY12 expression levels decrease in induced HMC3‐Progerin cells (Figure 3G).

### 3.3 Induced HMC3‐Progerin Cells Display Age‐Associated Transcriptomic Changes

**FIGURE 4:**
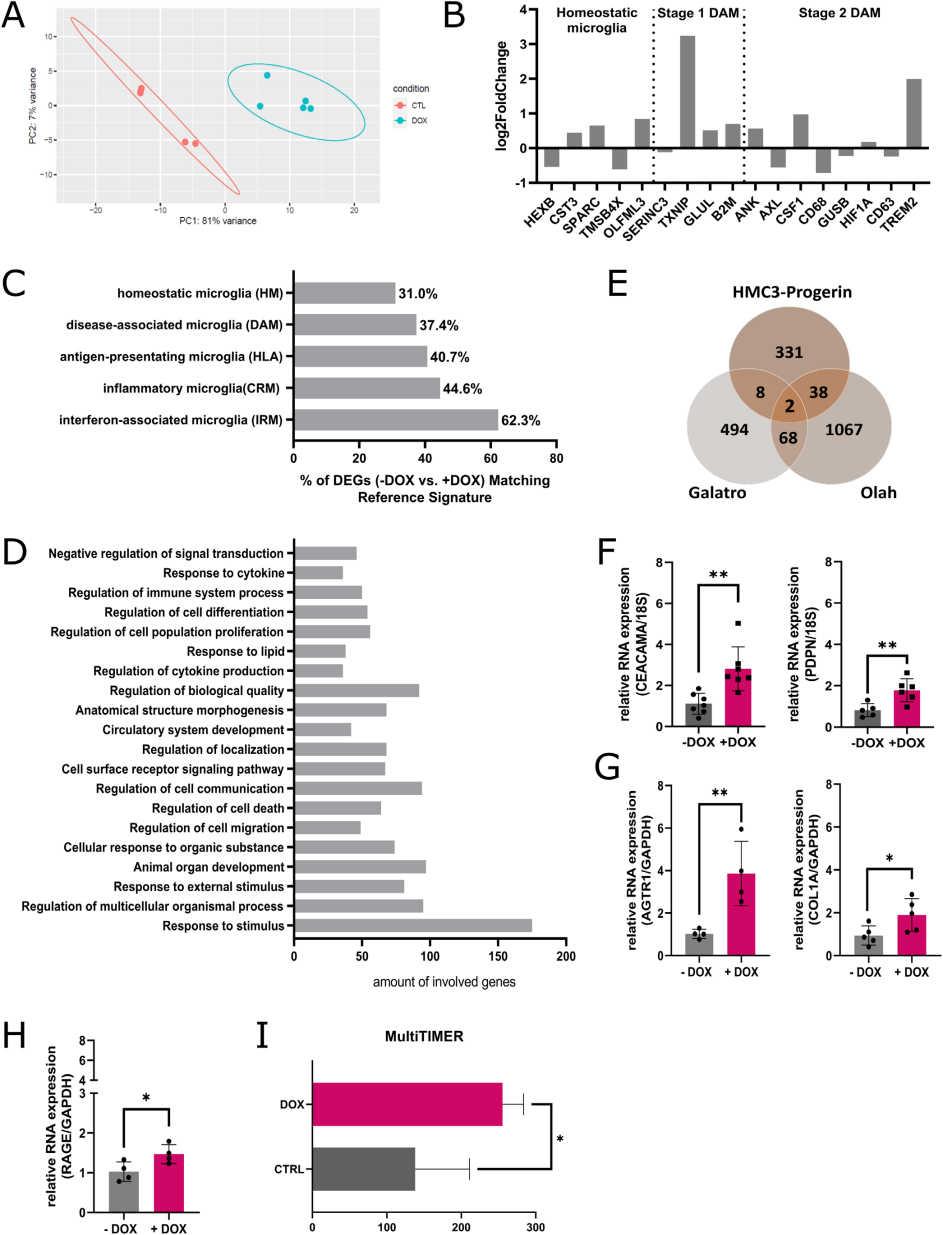
Transcriptomic profiling reveals aging‐associated signatures in HMC3‐Progerin microglia after 72 h of doxycycline induction. (A) Principal component analysis (PCA) of normalized RNA‐seq data demonstrates distinct clustering of +DOX and −DOX conditions. PC1 and PC2 account for 81% and 7% of the total variance, respectively, with each dot representing one biological replicate. (B) Log_2_ fold change of selected microglial marker genes, including homeostatic and disease‐associated microglia (DAM stage 1 and 2) markers, reveals a transcriptional shift towards a DAM‐like phenotype upon progerin induction. (C) Analysis with the Microglia Annotation Tool revealed that a subset of the DEGs identified by RNA sequencing (–Dox vs. +Dox) partially overlaps with established reference signatures defined by specific phenotype marker genes: 31.0% homeostatic (HM), 37.4% disease‐associated (DAM), 40.7% antigen‐presenting (HLA), 44.6% inflammatory (CRM), and 62.3% interferon‐associated (IRM) microglia. (D) Gene ontology (GO) enrichment analysis of differentially expressed genes (DEGs) highlights biological processes altered in induced (+DOX) HMC3‐Progerin cells. (E) Venn diagram shows DEG overlap with published human microglia datasets. A total of 2 genes (*CEACAM1*, *PDPN*) were shared across all datasets; 8 overlapped with Galatro et al. (2017), and 38 with Olah et al. (2018). (F–H) RT‐qPCR validation of selected DEGs confirms upregulation of *CEACAM1* and *PDPN* (F), *AGTR1* and *COL1A* (G), and *RAGE* (H) in induced (+DOX) HMC3‐Progerin relative to non‐induced (−DOX) controls. (I) Transcriptomic age estimation using the Multi‐Timer algorithm revealed a significant increase in the aging score in induced (+DOX) HMC3‐Progerin cells, indicating an accelerated transcriptomic aging profile. *All data are shown as mean ± SD; *p < 0.05, **p < 0.001, **p < 0.0001; unpaired student's *t*‐test.

Next, we wanted to assess how global transcriptome is altered after premature aging of HMC3 via progerin expression. Therefore, we performed RNA‐sequencing and compared gene expression of non‐induced to induced HMC3‐Progerin cells. Principal component analysis (PCA) of DEGs showed clear separation between non‐induced and induced HMC‐Progerin cells (Figure 4A), with PC1 accounting for 81% of the variance, indicating a strong effect of the premature aging via progerin expression. Biological replicates clustered tightly, reflecting high reproducibility. Next, we analyzed microglia specific core genes (Figure 4B) characterized by Keren‐Shaul et al. (2017). We found gene expression changes in genes that characterize DAMs especially in Stage 2. We further used the “microglia annotation tool” (Fumagalli et al. 2025), a bioinformatics resource to identify microglia cell states from high‐dimensional datasets. DEGs of +DOX compared to –DOX cells were directly compared to established reference signatures (coming from both xenotransplant human microglia and human postmortem datasets) of identified microglial cell states defined by specific marker genes. The results show that among all DEGs (‐DOX vs. +DOX), 62.3% overlapped with interferon‐associated, 44.6% with inflammatory, 40.7% with antigen‐presenting, 37.4% with disease‐associated, and only 31% with homeostatic microglia genes (Figures 4C, S4A). Next, we performed a Gene Ontology (GO) analysis. This revealed age‐related changes in RNA expression of genes involved in stimulus response (e.g., *MMP2*, *IGFBP5*, *CLDN1*), responses to external stimuli (e.g., *EDNRA*, *FOXG1*, *LONP1*), regulation of cell migration (e.g., *CD7*, *NGFR*, *CCL2*) as well as regulation of cell communication (e.g., *CYP26B1*, *CD74*, *CEACAM*) (Figure 4D). Comparison with published human aging datasets (Galatro et al. 2017; Olah et al. 2018) identified consistent dysregulation of CEACAM1 and PDPN, both implicated in senescence and vascular or stem cell aging (H. Y. Kim and Kim 2024; Kleefeldt et al. 2019). These genes were validated via qPCR and display a significant upregulation (Figure 4F). Furthermore, we found that genes (*COL1A*, *SERPINE1*, *F3*, *AGTR1*) that play an important role in the AGE‐RAGE axis (MacLean et al. 2021) are misregulated. Advanced glycation end‐products (AGEs) are a heterogeneous group of molecules that are formed to a greater extent in diseases such as inflammation, neurodegeneration or with increasing age (Bhattacharya et al. 2023; MacLean et al. 2021). We could confirm the results of the RNA‐sequencing via qPCR for *AGTR1* as well as *COL1A* (Figures 4G, S4B) and observed an upregulation in RNA expression of the receptor of AGEs (*RAGE*) (Figure 4H). Comparison with KEGG pathways revealed dysregulation of 32 nucleocytoplasmic transport‐related (hsa03013), 47 AGE‐RAGE pathway‐related (hsa04933) and 45 phagocytosis‐related genes (hsa04145), aligning with the impaired phagocytic function observed in induced HMC3‐Progerin cells (Table S4). Moreover, we detected an upregulation of *CPEB4*, *EIF2A* and *ZC3H12A*, key regulators involved in SG formation. Finally, analysis using the AgeClock MultiTimer revealed an upregulation in prematurely aged HMC3‐Progerin cells (Figure 4I) (Jung et al. 2023).

### 3.4 Senolytic treatment attenuates the age‐associated phenotype of HMC3‐Progerin

**FIGURE 5:**
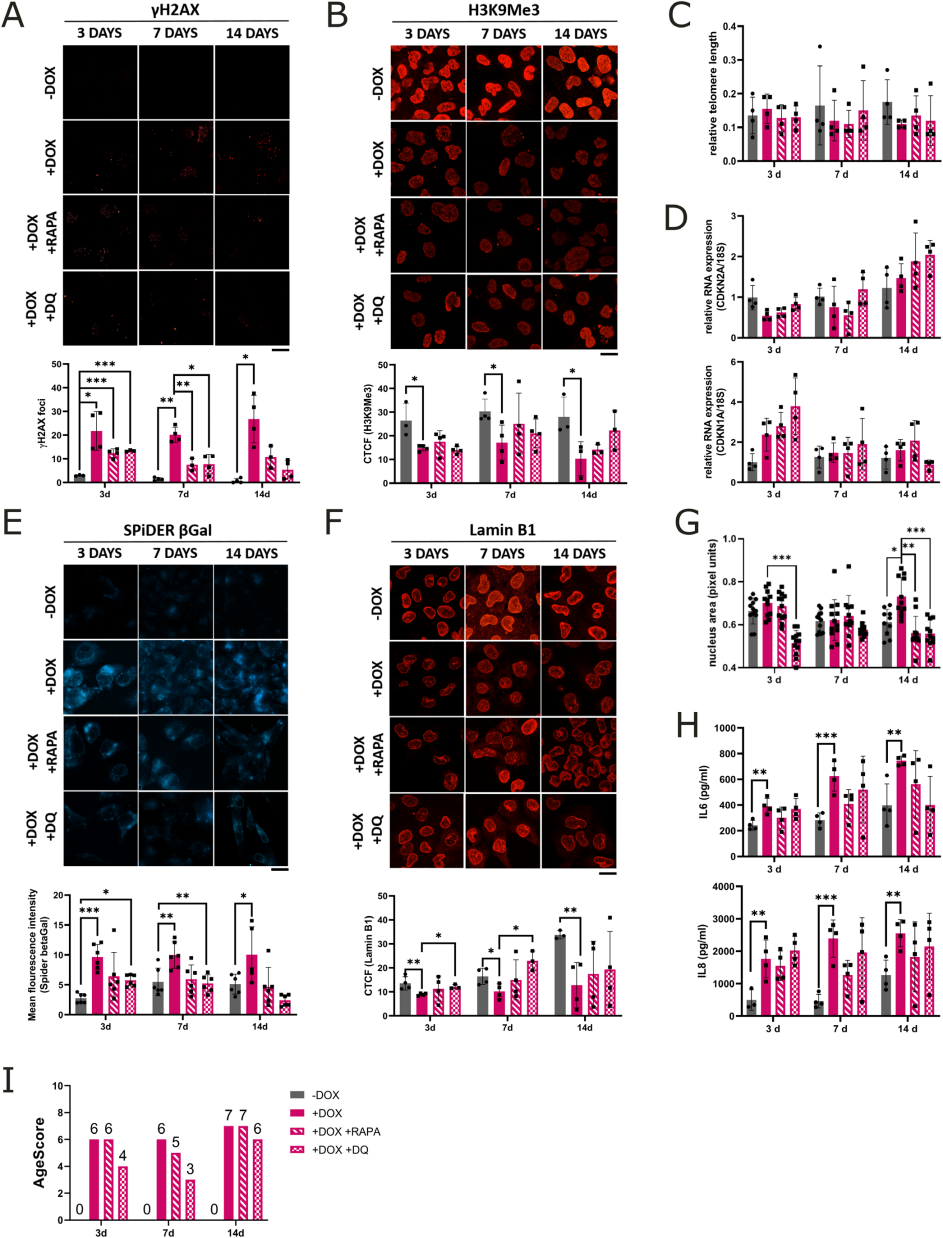
Senolytic treatment partially reverses aging‐associated phenotypes in HMC3‐Progerin microglia. HMC3‐Progerin cells were treated with 500 nM rapamycin (RAPA) or a combination of 200 nM dasatinib and 10 μM quercetin (DQ) for 3, 7, or 14 days. (A) γH2A.X foci, indicative of DNA damage, were significantly reduced after 3 and 7 days of senolytic treatment, but not at day 14. (B) H3K9Me3 expression remained reduced in all treatment conditions, indicating that this histone modification was not restored. (C) Telomere length, measured by MM‐qPCR, remained unchanged across all time points and treatments. (D) Expression levels of cell cycle regulators *CDKN2A* (p16) and *CDKN1A* (p21) measured via qRT‐PCR were unaffected by senolytic treatment. (E) SA‐βGal activity, assessed by Spider βGal staining (each n tested > 100 cells), revealed a reduction in senescent cells following DQ treatment at 3 and 7 days, but not at day 14. (F) Representative images and quantification of CTCF of Lamin B1 expression remained diminished across all treatment conditions and time points. (G) Nuclear area showed partial restoration only after 14 days of treatment. (H) Enzyme‐linked immunosorbent assay (ELISA) of conditioned medium of interleukin 6 (IL6) and interleukin 8 (IL8) secretion showed no significant decrease following RAPA or DQ treatment. (I) AgeScore analysis revealed a partial reduction at 3 and 14 days with DQ and at 7 days with both RAPA and DQ. *All data are presented as mean ± SD; statistical significance was determined by two‐way ANOVA with Sidak's post hoc test; *p < 0.05, **p < 0.001, **p < 0.0001; scale bar = 20 μm.

To investigate whether the age‐related phenotype in microglia could be reversed or attenuated, we first analyzed potential compounds capable of counteracting the observed gene expression changes. For this, we intersected our list of DEGs with the L1000CDS2 signature database to investigate whether the transcriptomic changes induced by prematurely aging might be reversed by FDA‐approved drugs. Using this analysis (Table S4), rapamycin emerged as a promising candidate. In addition, we also evaluated the combination of dasatinib and quercetin, as this senolytic cocktail is widely used to target senescent cells and modulate aging pathways, offering a potential way to lessen cellular and molecular signs of aging (Islam et al. 2023; Selvarani et al. 2021). We treated HMC3‐Progerin cells with rapamycin (500 nM) or a combination of dasatinib (200 nM) and quercetin (10 μM) for 3, 7, or 14 days. Excitingly, senolytic treatment was able to attenuate the progerin‐induced increase in the AgeScore. DNA damage improved after 3 and 7 days with both treatments (Figure 5A), but H3K9Me3 downregulation was unaffected (Figure 5B). Telomere attrition and cell cycle arrest showed no changes (Figure 5C,D). Dasatinib plus quercetin improved SA‐βGal and Lamin B1 levels at 3 and 7 days, but not after 14 days (Figure 5E,F). Nuclear morphology was rescued after 14 days by both treatments (Figure 5G), while the SASP phenotype remained unchanged (Figure 5H). AgeScore improvements appeared after 3 days with dasatinib plus quercetin, after 7 days with both treatments, and slightly after 14 days with dasatinib plus quercetin (Figure 5I). In control cells, only IL6 levels decreased after 7 days of rapamycin treatment (Figure S5). These findings suggest that senolytic treatment is capable of partially alleviating age‐associated cellular changes in microglia, although not all aging features respond equally to the intervention.

### 3.5 Premature Aging of Microglia Leads to a Defect in Nucleocytoplasmic Transport and Mislocalization of FUS Protein as Well as to Altered Stress Response

**FIGURE 6:**
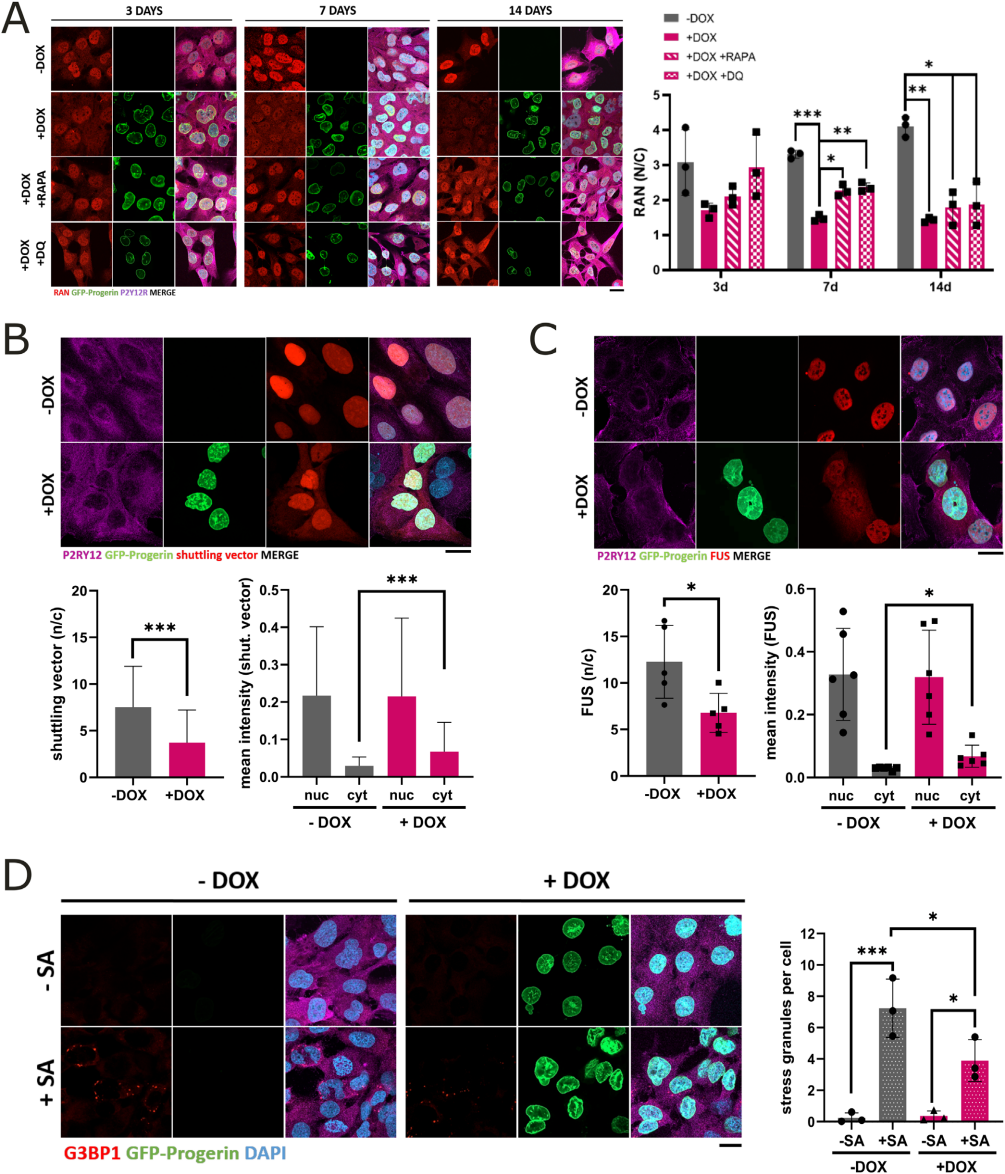
Nucleocytoplasmic transport (NCT) defect in HMC3‐Progerin cells leads to FUS mislocalization and altered stress response. (A) Immunofluorescence staining of RAN protein after 3, 7, and 14 days of doxycycline treatment, with or without senolytics (500 nM rapamycin [RAPA] or 200 nM dasatinib +10 μM quercetin [DQ]). In induced (+DOX) HMC3‐Progerin cells, RAN redistributed from the nucleus to the cytoplasm at days 7 and 14, as shown by the nucleus‐to‐cytoplasm intensity ratio (N/C). This effect was partially reversed after 7 days of senolytic treatment. (B) Representative images of the shuttling assay using a dtTomato reporter containing classical nuclear localization (NLS) and nuclear export (NES) sequences. Doxycycline‐induced (+DOX) HMC3‐Progerin cells showed impaired nuclear import and increased cytoplasmic accumulation compared to non‐induced (−DOX) controls, quantified by mean intensity ratios. (C) Immunofluorescence analysis of FUS protein revealed nuclear‐to‐cytoplasmic mislocalization in induced (+DOX) HMC3‐Progerin cells, as measured by intensity ratios between nucleus and cytoplasm. (D) Stress granule (SG) formation following exposure to 200 μM sodium arsenite (SA) for 1 h. Quantification showed a significant increase in SGs in both groups, with a more pronounced response in non‐induced (−DOX) controls. *All data are shown as mean ± SD; statistical analysis by two‐way ANOVA with Sidak's post hoc test (A) or one‐way ANOVA with Tukey's post hoc test/unpaired student's *t*‐test (B–D); *p < 0.05, **p < 0.001, ***p < 0.0001; scale bar = 50 μm.

The role of microglia aging itself on brain homeostasis and their influence on neurodegenerative processes is widely unknown. Because the impairment of NCT is a driver of senescence and often disrupted during neurodegenerative disease (Ding and Sepehrimanesh 2021), we wanted to know if prematurely aged microglia show impaired NCT. Therefore, we first analyzed the expression levels of Ras‐related nuclear protein (RAN) via IF (Figure 6A). Ran is a small GTPase that regulates the directionality of cargo transport between the nucleus and cytoplasm (Ding and Sepehrimanesh 2021). Fitting to above‐mentioned dysregulation of NCT in the KEGG analysis, we found a significant downregulation of RAN expression after 7–14 days of progerin expression in HMC3‐Progerin cells (Figure 6A), which could be restored by the treatment with rapamycin or the combination of dasatinib + quercetin.

To further validate a general disturbance of the NCT of induced HMC3‐Progerin cells, we transduced them with a dtTomato reporter with a classical nuclear localization sequence (NLS) and nuclear export signal (NES) via LAV. The results displayed a redistribution from the nucleus to the cytoplasm in induced compared to non‐induced HMC3‐Progerin cells (Figure 6B). Since cytoplasmic mislocalization of nuclear proteins is a hallmark of neurodegenerative diseases, particularly ALS, we performed IF staining of different RNA binding proteins (RBPs) associated with ALS and other neurodegenerative diseases (Figure 6C, S6), namely FUS, EWSR1 (Ewing sarcoma breakpoint region 1) and hnRNPA2B1 (heterogeneous nuclear ribonucleoprotein A2/B1) (H. J. Kim et al. 2013; Vance et al. 2013). Here, we were able to detect a significant mislocalization of FUS from the nucleus to the cytoplasm in induced HMC3‐Progerin cells; EWSR1 and hnRNPA2B1 were not affected.

Finally, we examined the microglial stress response (Figure 6D) via the formation of stress granules (SGs), cytoplasmic RNA granules composed of RBPs and untranslated mRNAs linked to the cytoplasmic metabolism of mRNA in response to cellular stress (Markmiller et al. 2018). Under normal conditions, SGs are dynamic structures that protect cells from stress, but prolonged stress causes them to mature into more stable complexes. Cytoplasmic mislocalization of RBPs typically triggers SG formation. Very recently, aging neurons were reported to suffer from chronic cellular stress that prevents SG formation (Rhine et al. 2025). Therefore, we assessed SG formation in prematurely aged microglia. For this, induced and non‐induced HMC3‐Progerin cells were incubated with sodium arsenite (SA) for 1 h at 37°C. Following treatment, both groups displayed a significant increase in SG formation. Of note, however, this response was blunted in prematurely aged microglia.

## 4 Discussion

Microglia, as guardians of the brain, play a vital role in maintaining healthy brain homeostasis. With increasing age, they change their phenotype, leading to impaired function of this cell type. The influence of aged microglia on the processes of age‐associated diseases as well as neurodegenerative processes is still largely unexplored.

In vitro cultures of aged human microglia are challenging to obtain. While iPSC models derived from aged donors might seem ideal for studying aging, the reprogramming process typically erases key aging markers (Roux et al. 2022), whereby a weak residual signature of the donor age can be observed (Lo Sardo et al. 2017). HMC3 are human microglia of fetal origin, which have been immortalized. Overexpression of progerin might offer an opportunity to induce aging. The combination of HMC3 cells with an inducible progerin system offers an attractive model, which combines controlled aging with scalability and decreased variability.

Progerin results from the production of a partially processed form of the nuclear fibril protein Lamin A and plays a crucial role in HGPS, a rare genetic disorder leading to premature aging (Scaffidi and Misteli 2006). Mechanistically, the expression of progerin leads to numerous morphological changes in the cell nuclei as well as disruptions in DNA replication, mitosis, and alterations in heterochromatin organization (Shumaker et al. 2006). Notably, progerin has also been identified in fibroblasts from healthy older donors, leading to HGPS‐like defects, including increased DNA damage, reduced expression of H3K9Me3, and telomere shortening (Scaffidi and Misteli 2006). In our previous research, we demonstrated that overexpressing progerin in fibroblasts from young donors activates various age markers, such as a decrease in Lamin B1 expression, activation of the SASP, and initiation of cell cycle arrest (Hartmann et al. 2023). Furthermore, inhibiting progerin expression in old, non‐HGPS donors enhances proliferative activity, increases H3K9Me3 expression, and reduces various senescence markers (e.g., IGFBP3, GADD45B) (Scaffidi and Misteli 2006). These findings suggest that progerin‐dependent mechanisms resemble those of normal aging. While not all aging processes can be fully represented by the Lamin A mutation, our model offers a new avenue to study the aging of human microglia. The overexpression of progerin in HMC3 enables the targeted induction of age‐related features, such as nuclear envelope abnormalities, increased genomic instability, and reduced cellular resilience. This more accurately mimics the cellular hallmarks of aging (López‐Otín et al. 2013). Consequently, overexpressing progerin in an immortalized model is suitable for investigating the molecular mechanisms underlying microglial aging and its role in neurodegenerative disease progression.

Inducible progerin expression in HMC3 microglia increases the AgeScore, a composite measure combining common aging markers into one single value. The AgeScore was developed to provide a quantitative and comparative tool to measure aging‐associated cellular responses under defined conditions, including but not limited to microglia and/or progerin expression and includes several age markers to depict aging processes more broadly. While weighting single items within the AgeScore is possible, it is not devoid of bias since the impact of each marker might vary across cell types and conditions. Thus, weighting factors were opted out. Transcriptomic data revealed a significant age‐associated shift in our microglia model fitting to increased AgeScore. Despite its simplicity, the AgeScore was able to catch these differences, but should be complemented by detailed marker analyses to capture aging's complexity.

Beside an increase in various age markers, our microglia aging model displayed a baseline activation. This was, however, not linked to a “hyperactivated” state, but to a dampened stress‐induced activation and reduction in the most important functions of this cell type: migration and phagocytosis. Previous studies have already shown that microglia exhibit these limitations with increasing age (Damani et al. 2011; Hefendehl et al. 2014). Hefendehl et al. observed a decrease in the phagocytosis of *E. coli* beads, whereby the internalization of neural debris was even increased. Functional impairments of aged microglia additionally include a reduced ability to take up amyloid‐β fibrils (Floden and Combs 2011) and reduced chemotaxis, motility, and migration in response to laser‐induced injury and extracellular ATP (Damani et al. 2011; Hefendehl et al. 2014). Microglia therefore appear to show a reduction in their functional capacity in addition to hyperactivation, which ultimately supports age‐associated declines and alters brain homeostasis. Furthermore, our findings demonstrate a reduction in SG formation capacity in induced HMC3‐Progerin cells upon acute SA‐induced stress. This appears counterintuitive given the cytoplasmic accumulation of the RBP FUS or that progerin expression is associated with elevated cellular stress levels, including oxidative and ER stress. However, this paradox can be reconciled by considering the phenomenon of stress response desensitization. Chronic exposure to cellular stress, such as that induced by sustained progerin expression, may blunt acute SG assembly through adaptive or maladaptive mechanisms, as previously observed in other models of persistent stress exposure (Rhine et al. 2025; Szewczyk et al. 2023). In addition, transcriptomic profiling of induced HMC3‐Progerin cells revealed dysregulation of SG‐related factors such as *CPEB4*, *EIF2A*, and *ZC3H12A*, suggesting a molecular basis for impaired stress granule formation. It is also conceivable that cytoskeletal alterations or RBP dysfunctions, both known consequences of progerin expression, hinder the dynamic assembly and maturation of SGs, despite an elevated basal stress load. In parallel, we observed a consistent and significant reduction in expression of the homeostatic microglial marker P2RY12 (Figure S3G), indicating a progressive shift away from a resting, homeostatic state. This is in line with previous descriptions of aged microglia acquiring a more reactive or disease‐associated phenotype. Our transcriptomic data further support this notion by showing an enrichment of gene signatures linked to interferon‐responsive and disease‐associated microglia profiles (Figure 4C). These findings underscore the complex reprogramming of microglia during aging, wherein basal activation coexists with selective impairments in classical stress response pathways and homeostatic maintenance. This dysregulation impairs important functions of microglia, such as phagocytosis and inflammation regulation, and promotes neurodegenerative processes (Wolf et al. 2017). In summary, while aged microglia exhibit elevated baseline levels of pro‐inflammatory cytokine secretion, their ability to respond to stressors is impaired.

RNA sequencing of prematurely aged HMC3‐Progerin cells revealed a distinct transcriptomic signature that mirrors several hallmarks of microglial aging. This includes a transcriptional reprogramming that mimics the immune activation and state transitions observed in aging and neurodegenerative disease. Notably, comparison with human datasets from aged microglia (Galatro et al. 2017; Olah et al. 2018) revealed overlapping expression changes. Moreover, GO enrichment analyses highlighted dysregulation in pathways related to cell migration, response to stimuli, and intercellular communication, processes essential to effective microglial surveillance and homeostasis. GO analysis further revealed misregulated genes like *COL1A*, *SERPINE1*, *F3*, and *AGTR1* involved in the AGE‐RAGE signaling pathway (Bhattacharya et al. 2023; MacLean et al. 2021). AGEs are glycated proteins or lipids that accumulate with age, contributing to age‐related diseases and degeneration (Bhattacharya et al. 2023). RAGE, their receptor, binds multiple ligands and influences microglial signaling, promoting inflammation and neuronal damage in AD models (Fang et al. 2010). It also disrupts microglial communication in ALS mice, shifting them to a harmful phenotype (MacLean et al. 2021). Our results show misregulation of AGE‐RAGE signaling and increased *RAGE* in aged microglia. Further studies are needed to clarify its role in microglial communication and aging phenotypes. Together, these findings underscore the utility of our HMC3‐Progerin model in capturing age‐associated microglial reprogramming across both functional and transcriptional levels and support its relevance for studying mechanisms of neuroinflammatory aging and disease.

With increasing age, there is also a downregulation of nuclear transport factors resulting in restricted protein transport between the nucleus and cytoplasm (Park et al. 2021). Additionally, NCT disruption is a hallmark of senescent cells (Park et al. 2021). Transcriptomic changes observed in HMC3‐Progerin cells included altered expression of genes involved in NCT. We indeed investigated and found a general disturbance of NCT in HMC3‐Progerin in comparison to the non‐induced control cells (Figure 6A–C). Beyond aging, alterations in NCT are observed during neurodegenerative processes as well. For instance, in many cases of ALS and frontotemporal dementia (FTD), RBPs such as FUS and TDP43 are increasingly mislocalized to the cytoplasm, where they can aggregate (Dormann et al. 2010). So far, this pathological mislocalization has been attributed to the diseased protein itself, either by mutations affecting the NLS/NES or disturbed interaction with import/export proteins (Dormann et al. 2010). In our microglial aging model, we identified a defect in NCT along with a mislocalization of FUS protein. This highlights that aging‐associated changes might contribute themselves to cytoplasmic accumulation of proteins, which might be of particular interest in sporadic neurodegenerative diseases. EWSR1 and hnRNPA2B1 have also been implicated in ALS, but these two RBPs did not exhibit defects in NCT (Figure S6). Although all three proteins share structural similarities as RBPs of the TET family, they differ in their nuclear import mechanisms and sensitivity to NCT disruption. FUS contains a well‐characterized PY‐type nuclear localization signal (PY‐NLS) and is highly dependent on Transportin‐1 mediated import, a pathway that is particularly vulnerable to disturbances in the RanGTP gradient or nuclear pore integrity (Khalil et al. 2024). In contrast, EWSR1 and hnRNPA2B1 may rely on partially redundant or more robust nuclear import mechanisms (e.g., Importin α/β) and have been reported to be more resistant to mild transport defects (Dormann et al. 2010; H. J. Kim et al. 2013). These differences likely explain why FUS, but not the other TET family members, responds to progerin‐induced stress and NCT disruption with mislocalization. Thus, FUS mislocalization in our model may serve as a sensitive marker for transport dysfunction and early pathological changes relevant to neurodegenerative disease contexts. Our findings suggest a general defect in the NCT of microglia that differentially affects specific proteins with age. This differential involvement may indicate that microglia play distinct roles in the pathophysiological cascades of various neurodegenerative diseases. Cytoplasmic mislocalization of FUS is normally associated with impaired proteostasis and increased SGs (Szewczyk et al. 2023). The finding of reduced SGs but increased cytoplasmic FUS protein might, however, suggest that aged microglia might even themselves suffer from proteotoxic stress and thus might aggravate proteotoxicity. Further studies are needed to explore this hypothesis in more detail.

Although overexpressing progerin in immortalized microglia offers a promising approach to modeling cellular aging, several limitations must be acknowledged. First, while progerin induces aging‐associated features such as nuclear morphological abnormalities and increased DNA damage, it might not fully recapitulate the multifactorial nature of physiological aging (López‐Otín et al. 2013). The exogenous overexpression of progerin may lead to non‐physiological levels that exaggerate certain phenotypes, potentially generating artifacts not observed in vivo. Moreover, the use of immortalized microglial cell lines, which inherently exhibit altered cell cycle regulation and gene expression compared to primary cells, might interfere with some classical age‐related processes, such as cell cycle arrest and thus limit the direct translatability of the results. Additionally, this model focuses predominantly on nuclear lamina alterations, thereby possibly underestimating other critical aspects of the aging process such as metabolic dysregulation, inflammatory signaling, and intercellular communication. These limitations highlight the need for complementary approaches to achieve a comprehensive understanding of microglial aging and its implications for neurodegeneration.

Nevertheless, we developed a human microglia in vitro model with controlled accelerating aging. We were able to induce premature aging in HMC3 cells through the expression of progerin. The prematurely aged HMC3‐Progerin cells exhibit activation of various age markers, along with functional impairment of the key microglial functions: migration and phagocytosis, but also reduced stress response. These results overlap with the age‐related transcriptomic changes in our study. We also found a defect in NCT in prematurely aged microglia, leading to the mislocalization of several proteins, including FUS, which is significantly implicated in FUS‐ALS and FUS‐FTD. This mislocalization may impair microglial function and potentially contribute to neurodegenerative processes. Future studies should further explore these findings. Nonetheless, the HMC3‐Progerin aging model offers a straightforward in vitro approach to investigate the mechanisms underlying human microglial aging.

## Author Contributions

Christiane Hartmann drafted the manuscript with the input of Andreas Hermann. Christiane Hartmann, Christina Haß, Muriel Knobloch, Maite Peters, Georgia Koromila, Franz Markert, Alexander Hartmann, Jessie Premereur, Kathrin Jäger, Laura Fumagalli, and Jette Abel did the experiments; Christiane Hartmann, Christina Haß, and Israel Barrantes did the analyses underlying the preparation of the tables and figures. Andreas Hermann, Michael Walter, Alexander Storch, Renzo Mancuso, and Georg Fuellen provided the main resources and funding. All authors approved the final version of the manuscript and made substantial, direct, and intellectual contributions to the work, and approved it for publication.

## Conflicts of Interest

The authors declare no conflicts of interest.

## Supporting information


**Figure S1:** Doxycycline treatment for 72 h does not induce cell damage in HMC3 cells. A, B: Quantification of cell growth (total cell number) and cell death (trypan blue‐positive cells) in HMC3 control and HMC3‐Progerin cells with or without doxycycline treatment over 72 h. Neither doxycycline treatment nor progerin overexpression affected cell proliferation or induced significant cell death. C: Quantification of γH2A.X foci in HMC3 CTRL in comparison to HMC3‐Progerin cells without or with doxycycline treatment. Doxycycline treatment did not increase DNA damage in HMC3 CTRL. Progerin expressing cells (+DOX) displayed however an increase in γH2A.X foci in comparison to controls (‐DOX). In contrast, doxycycline‐induced (+DOX) progerin expression led to a significant increase in γH2A.X foci. *All data are shown as mean ± SD; statistical significance was determined using one‐way ANOVA with Tukey's post hoc test; *p < 0.05, **p < 0.001, **p < 0.0001.
**Figure S2:** Analysis of cell cycle arrest markers and nuclear morphology in HMC3‐Progerin cells. A, B. Corrected total cell fluorescence (CTCF) quantification of p16 (A) and p21 (B) protein levels by immunofluorescence staining after 3, 7 and 14 days of doxycycline treatment. No significant differences were observed between induced (+DOX) and non‐induced (−DOX) HMC3‐Progerin cells at any time point. C. CTCF quantification of the proliferation marker Ki67 showed no change between +DOX and − DOX cells across all time points. D. Schematic illustration of solidity calculation. E. Quantification of nuclear solidity showed no significant difference between induced (+DOX) and non‐induced (‐DOX) HMC3‐Progerin cells. F. Schematic illustration of form factor calculation. G. Quantification of nuclear form factor revealed a significant reduction in induced (+DOX) HMC3‐Progerin cells after 3, 7 and 14 days, indicating changes in nuclear shape. *All data are presented as mean ± SD; statistical significance was determined by two‐way ANOVA with Sidak's post hoc test; *p < 0.05, **p < 0.001, **p < 0.0001; scale bar = 20 μm.
**Figure S3:** Analysis of cell cycle arrest and phagocytosis following Mitomycin C treatment. A–C: Cell cycle analysis by flow cytometry using propidium iodide staining in untreated and mitomycin C‐treated HMC3‐Progerin cells after 24 h (A) and 48 h (B). Treatment induced accumulation of cells in the G2/M phase. Quantification (C) shows a significant decrease in G0/G1 phase and an increase in G2/M phase cells after 48 h. D: Quantification of live and dead cells following 24 h and 48 h of mitomycin C treatment revealed no significant increase in cell death. E. Quantification of phagocytosis in induced (+DOX) HMC3‐Progerin cells showed partial restoration of phagocytic activity after 3, 7 and 14 days of rapamycin (RAPA) treatment. F. In non‐induced (‐DOX) HMC3‐Progerin cells, treatment with either rapamycin (RAPA) or the combination of dasatinib + quercetin (DQ) showed no significant effect on phagocytosis at any time point. G. CTCF quantification of P2RY12 protein expression by immunofluorescence staining in induced (+DOX) HMC3‐Progerin cells after 3, 7 and 14 days in comparison to non‐induced (‐DOX) controls. A significant reduction in P2RY12 expression was observed in induced (+DOX) HMC3‐Progerin cells at all time points. *All data are shown as mean ± SD; *p < 0.05, **p < 0.001, **p < 0.0001; statistical significance was determined by one‐way ANOVA with Tukey's post hoc test (C, E), paired student's t‐test (D), two‐way ANOVA with Sidak's post hoc test (F, G).
**Figure S4:** Induced (+DOX) HMC3‐Progerin cells exhibit transcriptomic changes associated with aging. A. Volcano plot highlighting the top 50 differentially expressed genes (DEGs) from the comparison of HMC3‐Progerin RNA‐seq data with microglial reference signatures using the Microglia Annotation Tool. The top 50 genes were visualized and color‐coded according to their predominant microglial cluster. Genes mapping to multiple clusters were assigned to only one cluster based on the highest log2 fold‐change. B. qRT‐PCR validation of selected DEGs (F3 and SERPINE1). All data are shown as mean ± SD; *p < 0.05, **p < 0.001, **p < 0.0001; unpaired student's t‐test.
**Figure S5:** Senolytic treatment has no effect on non‐induced HMC3‐Progerin. Non‐induced (−DOX) HMC3‐Progerin cells treated with 500 nM rapamycin or a combination of 200 nM dasatinib and 10 μM quercetin (DQ) showed no significant changes in the following parameters: A. DNA damage as measured by γH2A.X foci, B. H3K9Me3 expression, C. telomere length, D. p16 and p21 expression (cell cycle arrest marker), E. SA‐βGal activity, F. Lamin B1 expression, G. nuclea area, and H. secretion of IL6 and IL8 (SASP factors). *All data are shown as mean ± SD; statistical significance was assessed by two‐way ANOVA with Sidak's post hoc test; *p < 0.05, **p < 0.001, **p < 0.0001.
**Figure S6:** Analysis of nucleocytoplasmic transport (NCT) of EWSR1 and hnRNPA2B1 in prematurely aged HMC3‐Progerin. A, B. Representative images of IF staining of EWSR1 (A) and hnRNPA2B1 (B) in induced HMC3‐Progerin (+DOX) in comparison to non‐induced controls (‐DOX). No evidence of nuclear‐to‐cytoplasmic redistribution was observed in either protein, as assessed by nucleus‐to‐cytoplasm intensity ratios and mean fluorescence intensities in each compartment. *All data are presented as mean ± SD; statistical analysis by unpaired student's t‐test or one‐way ANOVA with Tukey's post hoc test; *p < 0.05, **p < 0.001, **p < 0.0001; scale bar = 50 μm.

**Table S1:** Primary and secondary antibodies used for Western Blot stainings.
**Table S2:** Primary and secondary antibodies used for immunfluorescent stainings.
**Table S3:** Primers used for qPCR.

**Table S4:** Results and comprehensive analysis of bulk RNA‐sequencing data between premature aged HMC3 and control cells.

## Data Availability

This RNAseq Illumina sequencing data from this work is available at the European Nucleotide Archive, under the project accession number PRJEB81801.
