# Supplementary material for: Prematurely Aged Human Microglia Exhibit Impaired Stress Response and Defective Nucleocytoplasmic Shuttling of ALS Associated FUS
Source: Aging Cell. 2025 Sep 19;24(11):e70232. doi: 10.1111/acel.70232 (PMC12610945; doi:10.1111/acel.70232)
Supplement: Supplementary file 1 — Figure S1: Doxycycline treatment for 72 h does not induce cell damage in HMC3 cells. A, B: Quantification of cell growth (total cell number) and cell death (trypan blue‐positive cells) in HMC3 control and HMC3‐Progerin cells with or without doxycycline treatment over 72 h. Neither doxycycline treatment nor progerin overexpression affected cell proliferation or induced significant cell death. C: Quantification of γH2A.X foci in HMC3 CTRL in comparison to HMC3‐Progerin cells without or with doxycycline treatment. Doxycycline treatment did not increase DNA damage in HMC3 CTRL. Progerin expressing cells (+DOX) displayed however an increase in γH2A.X foci in comparison to controls (‐DOX). In contrast, doxycycline‐induced (+DOX) progerin expression led to a significant increase in γH2A.X foci. *All data are shown as mean ± SD; statistical significance was determined using one‐way ANOVA with Tukey's post hoc test; *p < 0.05, **p < 0.001, **p < 0.0001. Figure S2: Analysis of cell cycle arrest markers and nuclear morphology in HMC3‐Progerin cells. A, B. Corrected total cell fluorescence (CTCF) quantification of p16 (A) and p21 (B) protein levels by immunofluorescence staining after 3, 7 and 14 days of doxycycline treatment. No significant differences were observed between induced (+DOX) and non‐induced (−DOX) HMC3‐Progerin cells at any time point. C. CTCF quantification of the proliferation marker Ki67 showed no change between +DOX and − DOX cells across all time points. D. Schematic illustration of solidity calculation. E. Quantification of nuclear solidity showed no significant difference between induced (+DOX) and non‐induced (‐DOX) HMC3‐Progerin cells. F. Schematic illustration of form factor calculation. G. Quantification of nuclear form factor revealed a significant reduction in induced (+DOX) HMC3‐Progerin cells after 3, 7 and 14 days, indicating changes in nuclear shape. *All data are presented as mean ± SD; statistical significance was determined by two‐way ANOVA wi [file ACEL-24-e70232-s002.docx]

**Supplementary Figure 1**


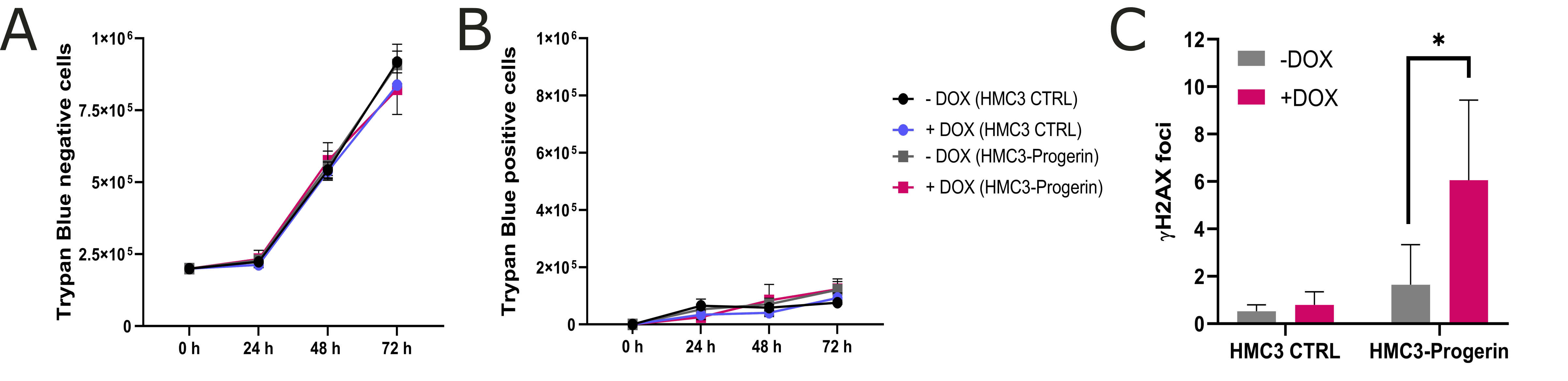


**Supplementary Figure 1: Doxycycline treatment for 72 h does not induce cell damage in HMC3 cells. A, B:** Quantification of cell growth (total cell number) and cell death (trypan blue-positive cells) in HMC3 control and HMC3-Progerin cells with or without doxycycline treatment over 72 h. Neither doxycycline treatment nor progerin overexpression affected cell proliferation or induced significant cell death. **C:** Quantification of γH2A.X foci in HMC3 CTRL in comparison to HMC3-Progerin cells without or with doxycycline treatment. Doxycycline treatment did not increase DNA damage in HMC3 CTRL. Progerin expressing cells (+DOX) displayed however an increase in γH2A.X foci in comparison to controls (-DOX). In contrast, doxycycline-induced (+DOX) progerin expression led to a significant increase in γH2A.X foci. *All data are shown as mean ± SD; statistical significance was determined using one-way ANOVA with Tukey’s post hoc test; *p < 0.05, **p < 0.001, **p < 0.0001.

**Supplementary Figure 2**


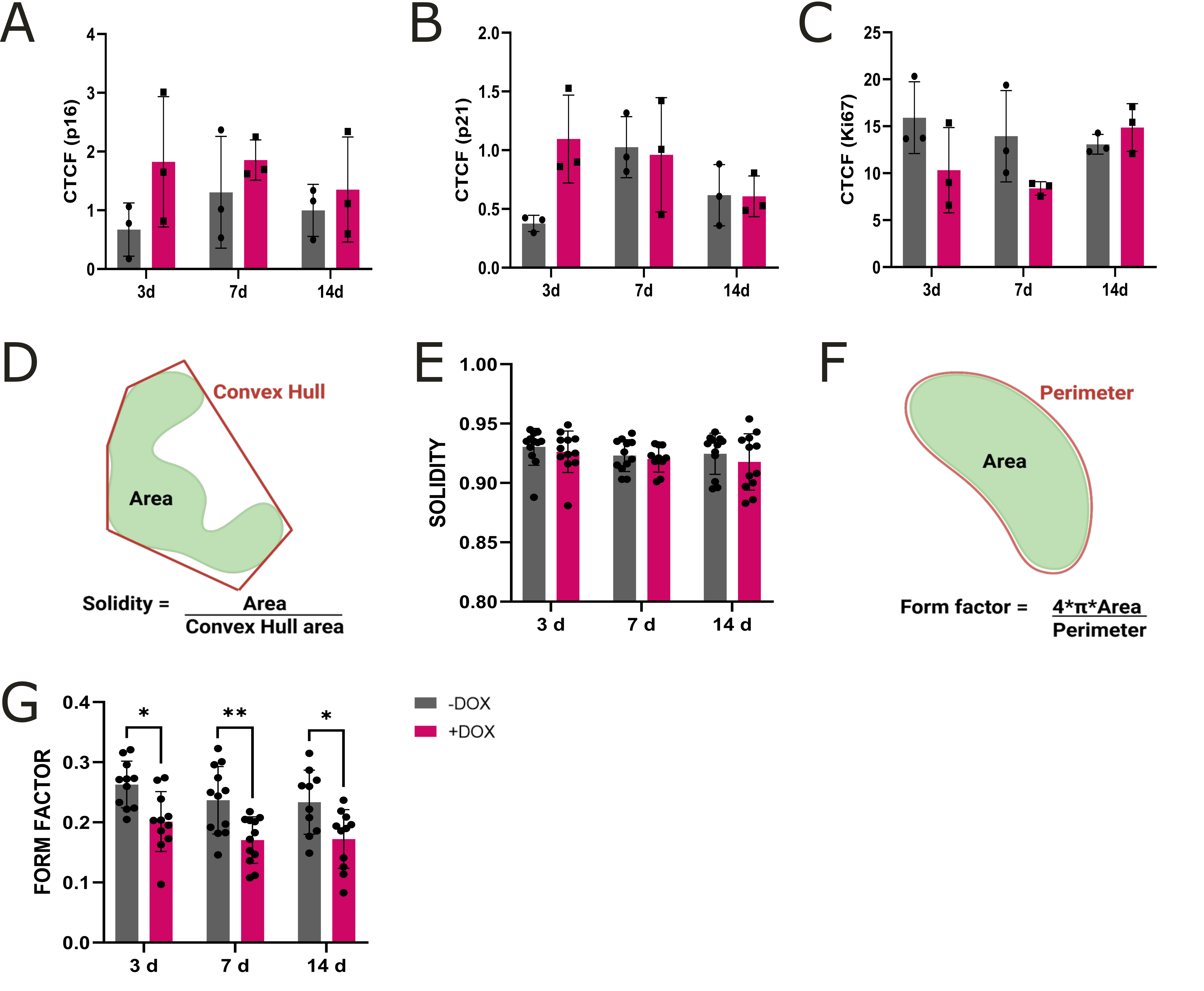


**Supplementary Figure 2: Analysis of cell cycle arrest markers and nuclear morphology in HMC3-Progerin cells. A-B.** Corrected total cell fluorescence (CTCF) quantification of *p16* (A) and *p21* (B) protein levels by immunofluorescence staining after 3, 7 and 14 days of doxycycline treatment. No significant differences were observed between induced (+DOX) and non-induced (−DOX) HMC3-Progerin cells at any time point. **C.** CTCF quantification of the proliferation marker Ki67 showed no change between +DOX and −DOX cells across all time points. **D.** Schematic illustration of solidity calculation. **E.** Quantification of nuclear solidity showed no significant difference between induced (+DOX) and non-induced (-DOX) HMC3-Progerin cells. **F.** Schematic illustration of form factor calculation. **G.** Quantification of nuclear form factor revealed a significant reduction in induced (+DOX) HMC3-Progerin cells after 3, 7 and 14 days, indicating changes in nuclear shape. *All data are presented as mean ± SD; statistical significance was determined by two-way ANOVA with Sidak’s post hoc test; *p < 0.05, **p < 0.001, **p < 0.0001; scale bar = 20 µm.

**Supplementary Figure 3**


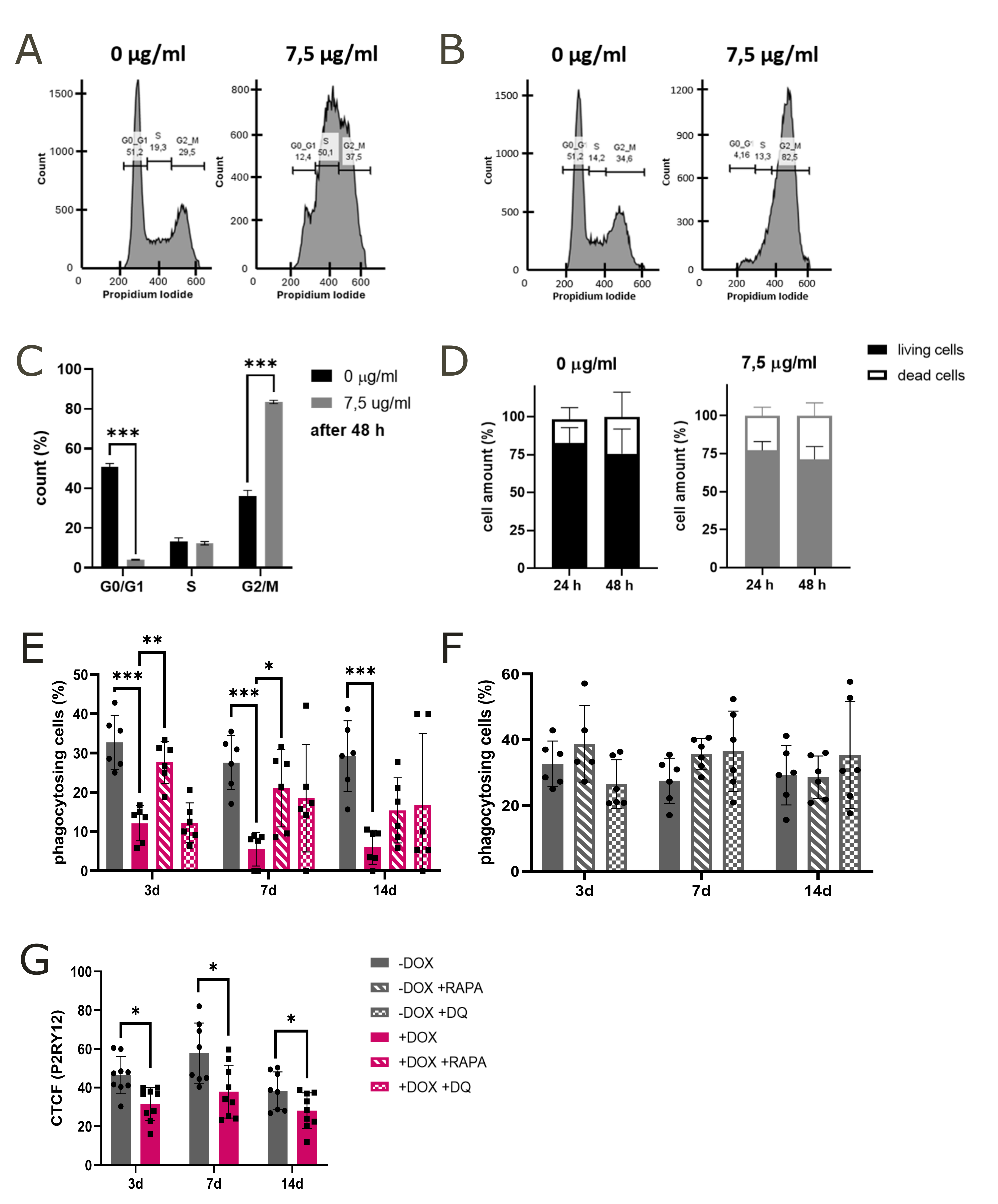


**Supplementary Figure 3: Analysis of cell cycle arrest and phagocytosis following Mitomycin C treatment. A-C:** Cell cycle analysis by flow cytometry using propidium iodide staining in untreated and mitomycin C-treated HMC3-Progerin cells after 24 h (A) and 48 h (B). Treatment induced accumulation of cells in the G2/M phase. Quantification (C) shows a significant decrease in G0/G1 phase and an increase in G2/M phase cells after 48 h. **D:** Quantification of live and dead cells following 24 h and 48 h of mitomycin C treatment revealed no significant increase in cell death. **E.** Quantification of phagocytosis in induced (+DOX) HMC3-Progerin cells showed partial restoration of phagocytic activity after 3, 7 and 14 days of rapamycin (RAPA) treatment. **F.** In non-induced (-DOX) HMC3-Progerin cells, treatment with either rapamycin (RAPA) or the combination of dasatinib + quercetin (DQ) showed no significant effect on phagocytosis at any time point. **G.** CTCF quantification of P2RY12 protein expression by immunofluorescence staining in induced (+DOX) HMC3-Progerin cells after 3, 7 and 14 days in comparison to non-induced (-DOX) controls. A significant reduction in P2RY12 expression was observed in induced (+DOX) HMC3-Progerin cells at all time points. *All data are shown as mean ± SD; *p < 0.05, **p < 0.001, **p < 0.0001; statistical significance was determined by one-way ANOVA with Tukey’s post hoc test (C, E), paired student’s t-test (D), two-way ANOVA with Sidak’s post hoc test (F, G).**Supplementary Figure 4**


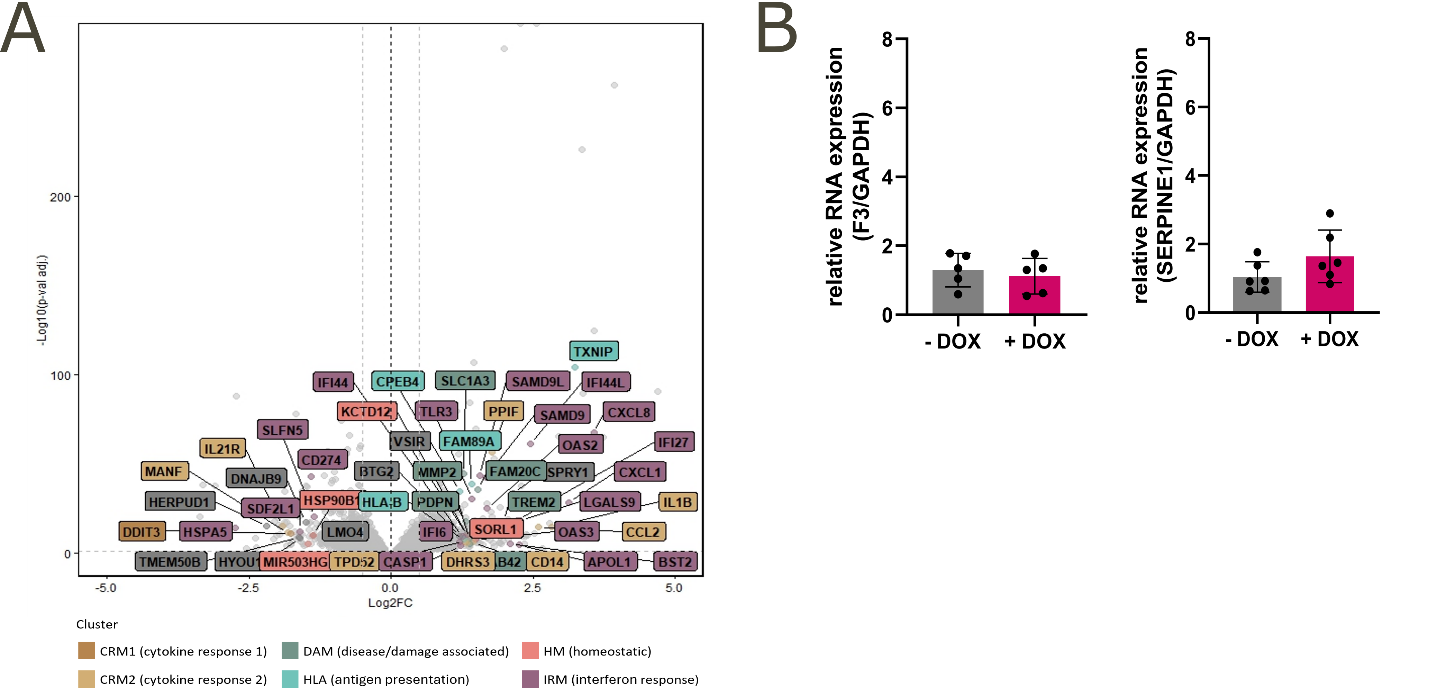


**Supplementary Figure 4: Induced (+DOX) HMC3-Progerin cells exhibit transcriptomic changes associated with aging.** **A.** Volcano plot highlighting the top 50 differentially expressed genes (DEGs) from the comparison of HMC3-Progerin RNA-seq data with microglial reference signatures using the Microglia Annotation Tool. The top 50 genes were visualized and color-coded according to their predominant microglial cluster. Genes mapping to multiple clusters were assigned to only one cluster based on the highest log2 fold-change. **B.** qRT-PCR validation of selected DEGs (F3 and SERPINE1). All data are shown as mean ± SD; *p < 0.05, **p < 0.001, **p < 0.0001; unpaired student’s t-test.

**Supplementary Figure 5**


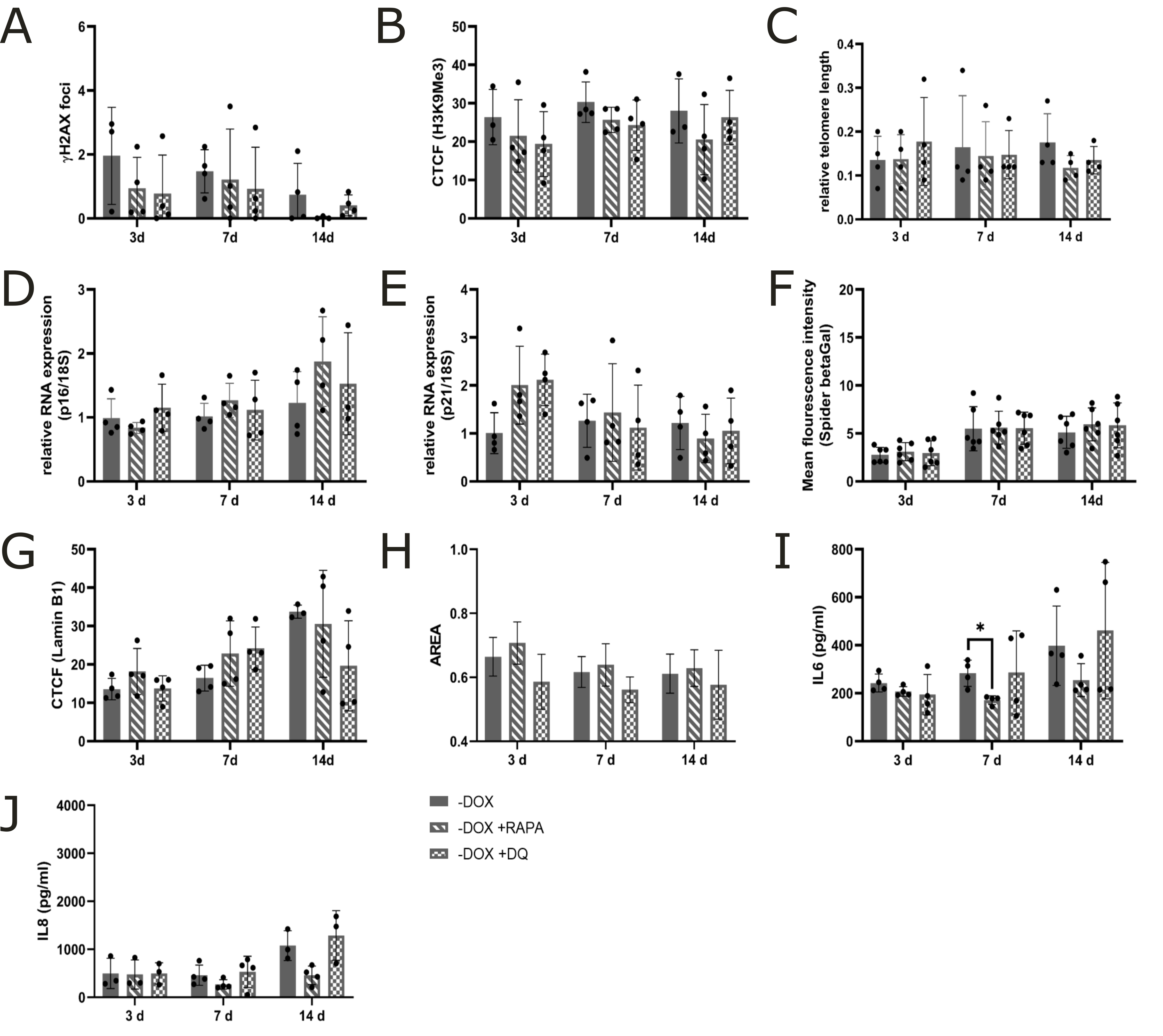


**Supplementary Figure 5: Senolytic treatment has no effect on non-induced HMC3-Progerin.** Non-induced (−DOX) HMC3-Progerin cells treated with 500 nM rapamycin or a combination of 200 nM dasatinib and 10 μM quercetin (DQ) showed no significant changes in the following parameters: **A.** DNA damage as measured by γH2A.X foci, **B.** H3K9Me3 expression, **C.** telomere length, **D.** p16 and p21 expression (cell cycle arrest marker), **E.** SA-βGal activity, **F.** Lamin B1 expression, **G.** nuclea area, and **H.** secretion of IL6 and IL8 (SASP factors). *All data are shown as mean ± SD; statistical significance was assessed by two-way ANOVA with Sidak’s post hoc test; *p < 0.05, **p < 0.001, **p < 0.0001.

**Supplementary Figure 6**


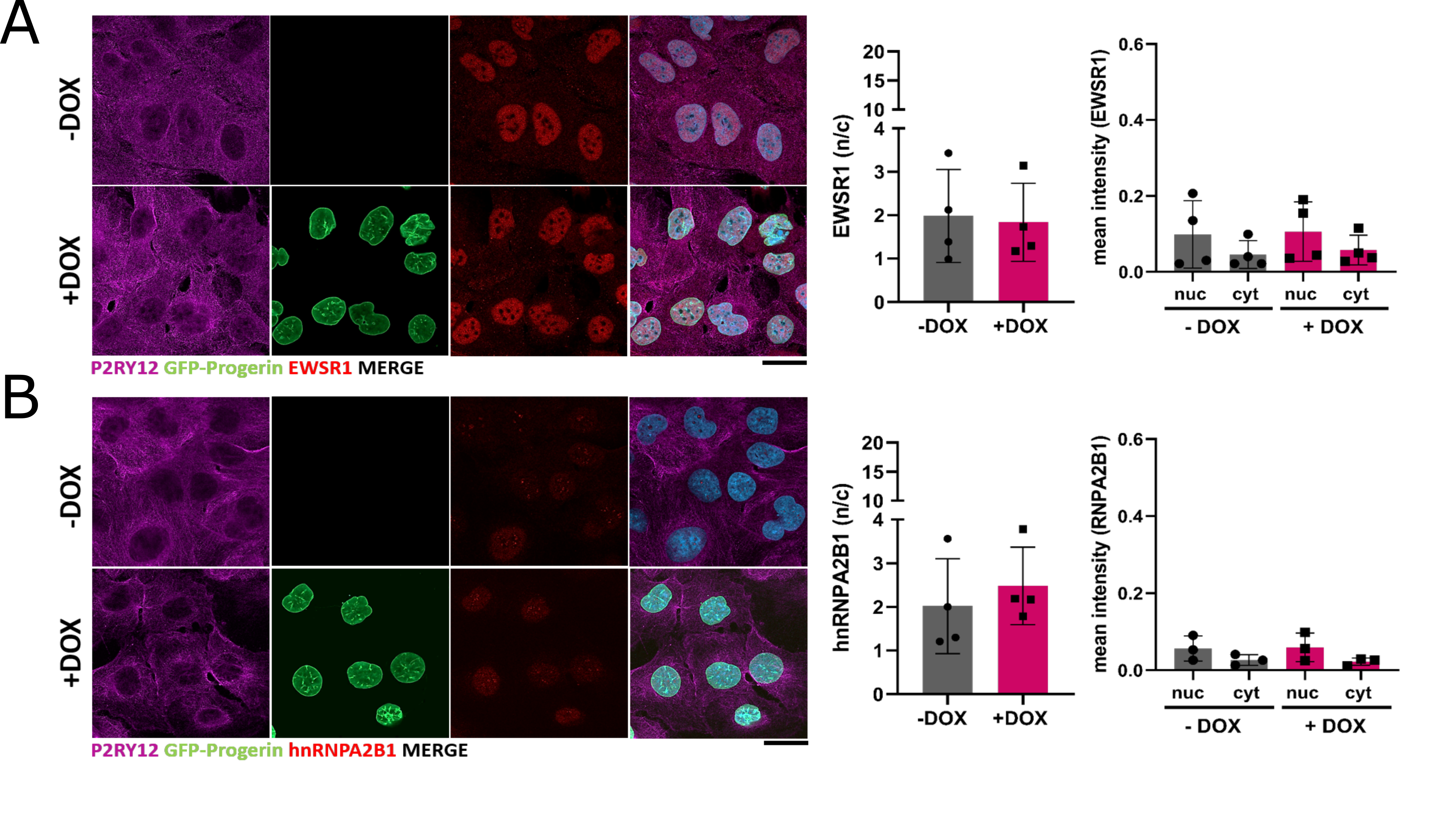


**Supplementary Figure 6: Analysis of nucleocytoplasmic transport (NCT) of EWSR1 and hnRNPA2B1 in prematurely aged HMC3-Progerin. A-B.** Representative images of IF staining of EWSR1 (A) and hnRNPA2B1 (B) in induced HMC3-Progerin (+DOX) in comparison to non-induced controls (-DOX). No evidence of nuclear-to-cytoplasmic redistribution was observed in either protein, as assessed by nucleus-to-cytoplasm intensity ratios and mean fluorescence intensities in each compartment. *All data are presented as mean ± SD; statistical analysis by unpaired student’s t-test or one-way ANOVA with Tukey’s post hoc test; *p < 0.05, **p < 0.001, **p < 0.0001; scale bar = 50 µm.
