# Supplementary material for: Prematurely Aged Human Microglia Exhibit Impaired Stress Response and Defective Nucleocytoplasmic Shuttling of ALS Associated FUS
Source: Aging Cell. 2025 Sep 19;24(11):e70232. doi: 10.1111/acel.70232 (PMC12610945; doi:10.1111/acel.70232)
Supplement: Supplementary file 2 — Table S1: Primary and secondary antibodies used for Western Blot stainings. Table S2: Primary and secondary antibodies used for immunfluorescent stainings. Table S3: Primers used for qPCR. [file ACEL-24-e70232-s003.docx]

| Antibody | Company and order number | Dilution |
| --- | --- | --- |
| *Anti-Lamin A + C antibody [JOL2]* | abcam, #ab40567 | 1:200 |
| *Anti-GAPDH antibody [EPR16891]* | abcam, #ab181602 | 1:10.000 |
| *Goat anti-Mouse IgG (H+L) Cross-Adsorbed Secondary Antibody* *DyLight™ 680* | Thermo Fisher Scientific, #35519 | 1:5.000 |
| *Goat anti-Rabbit IgG (H+L) Cross-Adsorbed Secondary Antibody, DyLight™ 800* | Thermo Fisher Scientific, #SA5-10036 | 1:5.000 |

**Supplementary Table 1:** Primary and secondary antibodies used for Western Blot stainings.

**Supplementary Table 2:** Primary and secondary antibodies used for immunfluorescent stainings.

| Antibody | Company and order number | Dilution |
| --- | --- | --- |
| *Anti-phospho-Histone H2A.X (Ser139)* | Millipore, #05-636-25ug | 1:500 |
| *Anti-Histone H3 (di methyl K9),* | abcam, #ab1220 | 1:500 |
| *Anti-Lamin B1* | abcam, #ab16048 | 1:500 |
| *Anti-P2Y12 Receptor* | Alomone labs, #APR-020-GP | 1:200 |
| *Anti-FUS* | NOVUS biologicals, #NB100-2599 | 1:200 |
| *Anti-EWSR1* | Thermo Fisher Scientific, #MA5-24791 | 1:1.000 |
| *Anti-hnRNAP2a* | abcam, #ab31645 | 1:250 |
| *Anti-RAN* | Thermo Fisher Scientific, #PA5-79913 | 1:100 |
| *Anti-Ki67* | abcam, #ab15580 | 1:500 |
| *Anti-p16INK4a* | Thermo Fisher Scientific, #MA5-17142 | 1:250 |
| *Anti-p21* | Thermo Fisher Scientific, # MA1-33926 | 1:250 |
| *Goat anti-Mouse IgG (H + L) Highly Cross-Adsorbed Secondary Antibody* | Invitrogen, #A-21236 | 1:500 |
| *Goat anti-Rabbit IgG (H + L) Highly Cross-Adsorbed Secondary Antibody* | Invitrogen, #A32740 | 1:500 |
| *Goat anti-Guinea Pig IgG (H+L) Highly Cross-Adsorbed Secondary Antibody* | Invitrogen, #A-21450 | 1:500 |

| GENE NAME | Forward Primer (5’-3’) | Reverse Primer (5’-3’) |
| --- | --- | --- |
| *CDKN1A* | GAC​ACC​ACT​GGA​GGG​TGA​CT | CAGGTCCACATGGTCTTCCT |
| *CDKN2A* | CTC​GTG​CTG​ATG​CTA​CTG​AGG​A | GGTCGGCGCAGTTGGGCTCC |
| *PROGERIN* | ACTGCAGCAGCTCGGGG | GGCTCTGGGCTCCTGAGCC |
| *AGTR1* | CAGCGTCAGTTTCAACCTGTACG | AGGAGCTACTGCTCCACCTTCT |
| *F3* | CAGAGTT CACACCTTACCTGGAG | GTTGTTCCTTCTGACTAAAGTCCG |
| *SERPINE1* | CTCATCAGCCACTGGAAAGGCA | GACTCGTGAAGTCAGCCTGAAAC |
| *COL1A2* | CCTGGTGCTAAAGGAGAAAGAGG | ATCACCACGACTTCCAGCAGGA |
| *RAGE* | CACCTTCTCCTGTAGCTTCAGC | AGGAGCTACTGCTCCACCTTCT |
| *TNFalpha* | CTCTTCTGCCTGCTGCACTTTG | ATGGGCTACAGGCTTGTCACTC |
| *IL6* | TACCACTTCACAAGTCGGAGGC | CTGCAAGTGCATCATCGTTGTTC |
| *CXCL10* | CCTGCATCAGCATTAGTAATCAACC | TGGATTCAGACATCTCTTCTCACC |
| *TGFbeta* | CAGTCACCATAGCAACACTC | CCTGGCCTGAACTACTATCT |
| *ARG1* | GGGCTACTCTCAGGATTAGAT | GCAGGTGACTTTGGCTACAAGC |
| *TREM2* | TCTGAGAGCTTCGAGGATGC | GGGGATTTCTCCTTCAAGA |
| *CEACAM* | CACGCCAATAACTCAGTCACTGG | TTGTGGAGCAGGTCAGGTTCAC |
| *PDPN* | GTGCCGAAGATGATGTGGTGAC | GGACTGTGCTTTCTGAAGTTGGC |
| *GAPDH* | GTCTCCTCTGACTTCAACAGCG | ACCACCCTGTTGCTGTAGCCAA |
| *18S* | ACC CGTTGAACCCCATTCGTGA | GCCTCACTAAACCATCCAATCGG |
| *Telg/Telc* | ACACTAAGGTTTGGGTTTGGGTTTGGGTTTGGGTTAGTGT | TGTTAGGTATCCCT ATCCCTATCCCTATCCCTATCCCTAAC |
| *Albu/Albd* | CGGCGGCGGGCGGCGCGGGCTGGGCGGAAATGCTGCACAGA ATCCTTG | GCCCGGCCCGCCGCGCCCGTCCCGCCGGAAAAGCATGGTCGCCTGTT |

**Supplementary Table 3:** Primers used for qPCR.
